# Two-step approach for assessing the health effects of environmental chemical mixtures: application to simulated datasets and real data from the Navajo Birth Cohort Study

**DOI:** 10.1186/s12940-019-0482-6

**Published:** 2019-05-09

**Authors:** Li Luo, Laurie G. Hudson, Johnnye Lewis, Ji-Hyun Lee

**Affiliations:** 1 Department of Internal Medicine, MSC10-5550, 1 University of New Mexico, Albuquerque, NM 87131 USA; 2 University of New Mexico Comprehensive Cancer Center, Albuquerque, NM USA; 3 Department of Pharmaceutical Sciences, College of Pharmacy, University of New Mexico, Albuquerque, NM USA; 4 Community Environmental Health Program, College of Pharmacy, University of New Mexico, Albuquerque, NM USA; 5 Present Address: Division of Quantitative Sciences, University of Florida Health Cancer Center; Department of Biostatistics, University of Florida, Gainesville, Florida USA

**Keywords:** Chemical mixtures, Two-step approach, Random Forest, Adaptive lasso

## Abstract

**Background:**

There is increasing interest in examining the consequences of simultaneous exposures to chemical mixtures. However, a consensus or recommendations on how to appropriately select the statistical approach analyzing the health effects of mixture exposures which best aligns with study goals has not been well established. We recognize the limitations that existing methods have in effectively reducing data dimension and detecting interaction effects when analyzing chemical mixture exposures collected in high dimensional datasets with varying degrees of variable intercorrelations. In this research, we aim to examine the performance of a two-step statistical approach in addressing the analytical challenges of chemical mixture exposures using two simulated data sets, and an existing data set from the Navajo Birth Cohort Study as a representative case study.

**Methods:**

We propose to use a two-step approach: a robust variable selection step using the random forest approach followed by adaptive lasso methods that incorporate both dimensionality reduction and quantification of the degree of association between the chemical exposures and the outcome of interest, including interaction terms. We compared the proposed method with other approaches including (1) single step adaptive lasso; and (2) two-step Classification and regression trees (CART) followed by adaptive lasso method.

**Results:**

Utilizing simulated data sets and applying the method to a real-life dataset from the Navajo Birth Cohort Study, we have demonstrated good performance of the proposed two-step approach. Results from the simulation datasets indicated the effectiveness of variable dimension reduction and reliable identification of a parsimonious model compared to other methods: single-step adaptive lasso or two-step CART followed by adaptive lasso method.

**Conclusions:**

Our proposed two-step approach provides a robust way of analyzing the effects of high-throughput chemical mixture exposures on health outcomes by combining the strengths of variable selection and adaptive shrinkage strategies.

**Electronic supplementary material:**

The online version of this article (10.1186/s12940-019-0482-6) contains supplementary material, which is available to authorized users.

## Background

Environmental chemical exposures often occur in mixtures and to our knowledge there is no consensus or established recommendations on how to appropriately select the statistical approach which best aligns with study goals assessing the health effects of mixture exposures [1–3]. The analytical challenges of complex mixtures are multifold. First, as humans are routinely exposed to multiple chemicals simultaneously or sequentially, high dimensionality of environmental chemical exposure data is common. Second, the toxicity of individual chemicals may depend on their interactions with other chemicals. Third, the nonlinear dose-response relationship is not commonly considered due to the difficult interpretation of multidimensional interactions. Fourth, statistical methods which appropriately incorporate the multicollinearity among chemicals have not been extensively investigated. Lastly, with the existence of multiple metal exposures along with the interactions among them, the statistical power for identification of the interaction effects is often low considering the large number of pairwise interactions.

To accommodate the analytical challenges in evaluating the health effects of the complex multiple interacting chemical mixture exposures, we propose to use a two-step approach: a robust variable selection step using the random forest approach followed by adaptive lasso methods. We expect this two-step approach incorporates both dimensionality reduction and quantification of the degree of association between the chemical exposures and the outcome of interest, including interaction terms. We will apply the proposed approach to analyze a high-dimensional data set from an ongoing study in our program: the Navajo Birth Cohort Study (NBCS). The NBCS is a prospective epidemiologic study designed to investigate the relationship between abandoned uranium mine (AUM)-related metal exposures, birth outcomes, and early developmental delays on the Navajo Nation. Previous studies have reported evidence on the link between AUM-related metal exposures and health outcomes [4, 5]. The issue is of particular concern on the Navajo Nation where more than 500 AUMs remain as a legacy of Cold War mining [6]. Exposure of community members to metal mixtures in AUM waste may contribute to diseases including hypertension, diabetes, and kidney disease [4, 5, 7]; arsenic and cadmium may damage the kidneys [8]; arsenic exposure may lead to retention of DNA damage by inhibiting DNA repair or directly causing DNA damage through oxidative damage [9, 10]; while the children’s exposures do not necessarily depend on the parents’ exposure in communities residing near abandoned mines, the parents’ exposure to toxic metals may have adverse effects on children’s health and developmental outcomes [11–13]. Considering the proximity of Navajo community members to AUMs, it is of importance to systematically investigate the health impact of simultaneous exposures to multiple metals.

The objective of this study is to utilize and test the performance of the proposed two-step statistical approach to address the analytical challenge in chemical mixture exposure studies, specifically, the high dimensional interacting complex chemical mixtures. We will compare the performance of the proposed approach to other methods using multiple simulated data sets. We will also apply this approach to examine the relationship between exposures to environmental metal mixtures and biomarkers of oxidative stress in the NBCS participants.

### Motivation

While there is increasing interest in examining the simultaneous exposures to chemical mixtures and rising concerns on the limited statistical approaches for handling chemical mixtures, it is our view that there is no consensus on the state-of-the-art statistical practices to analyze those data, or evaluation and recommendations of approach performance relative to dataset characteristics.

### Review of existing statistical approaches and their limitations

Recognizing the importance and analytical challenges in determining the health effects of complex mixture exposures, the National Institute of Environmental Health Sciences (NIEHS) convened a workshop with a special focus on the statistical approaches (“Statistical Approaches for Assessing Health Effects of Environmental Chemical Mixtures in Epidemiology Studies”) [1], which brought together multidisciplinary experts including epidemiologists, statisticians, and toxicologists. Participants applied various statistical methods to two simulated data sets assuming certain exposure–response relationships and one real-world data set containing human health data and relevant mixtures, and compared the analysis results at the workshop. The meeting organizers shared the lessons learned from learned the innovative workshop [1] and reported the observed considerable variability across different methods for the given datasets. The analysis results from many methods were more divergent and less aligned with the truth for the more complex simulated dataset provided by the workshop. The ability to detect interaction effects between exposures largely differed among the various approaches. The conclusion of the workshop is that there is no consensus on the state-of-the-art statistical methods for appropriate analysis of chemical mixtures as pointed out by the organizing committee and NIEHS scientists [1].

The statistical methods presented at the workshop can be classified into four major categories: 1) Classic linear regression; 2) Classification and prediction; 3) Variable selection and shrinkage strategies; 4) Exposure-response surface estimation [1]. While a fraction of the methods (21%) were Bayesian approaches, the majority of the presented methods were frequentist approaches. Each individual approach has its own limitations in determining the relationship between health outcomes with environmental chemical mixture exposures while appropriately accounting for issues such as multicollinearity, high dimensionality, interactions and non-linear dose response relationships.

It is worth noting that in order to overcome the limitation of individual single approaches, a few of the presented abstracts at the workshop employed combinations of two or more methods including 1) graphical visualization plus structural equation models; 2) a four-step procedure combining: screening, data transformation/combination, variable selection and model verification; 3) a two-step procedure of using prioritization of interactions followed by least absolute shrinkage and selection operator (lasso) approach: 4) principal component analysis plus regression strategies; 5) tree-based screening such as CART with a lasso approach. There are some limitations in the methods utilized in the combination approaches. First, screening based on correlations does not account for the interaction effects between exposures on the outcome variable [14]; second, regular regression approaches do not handle multicollinearity effectively [15, 16]; third, prioritizing based on interaction effects may bias the estimation of main effects [17]; fourth, the principal component regression coefficients are difficult to interpret, and therefore do not separate out the most important exposures [18, 19].

We note that tree-based and lasso approaches have been demonstrated to have appealing features that allow handling the analytical challenges of the chemical mixture data as presented in the workshop, and these methods are also increasingly employed in the field of high dimensional data analysis [20–25]. Motivated by a recent report which used combined CART and variable selection methods to analyze multiple pollutants and their interactions [3], we propose to use an improved two-step procedure of combining the random forest (RF) [25, 26] with adaptive lasso [27] approaches. The RF is used for reducing the dimensions of the multiple metal exposures in the first step, and the adaptive lasso approach is used in the second step for quantifying the association between the exposures and the outcome, including interaction terms. We expect that the proposed approach will overcome the limitations of CART (e.g. overfitting) and regular lasso approaches (e.g. bias in coefficient estimates). The RF method is a machine learning approach which effectively handles datasets with many more variables than subjects, captures nonlinear relationships between predictor variables, effectively handles missing values, and produces more robust results that are insensitive to outlier effects [28]. The adaptive lasso method is a modified version of lasso developed by Zou [27] that enjoys a very nice oracle property, i.e., it performs as well as if the true underlying model was given in advance.

### Motivating dataset from the NBCS

The Navajo Nation in the Four Corners region of the southwestern US covers a land area equivalent to the state of West Virginia, ~ 27,000 sq. mi. It is the largest US reservation, covering parts of Arizona, New Mexico and Utah, and the largest single-affiliation Native American tribe in the country [29]. The 2010 U.S. census reported 47% of the enrolled Navajo tribal population, or approximately 160,000 Navajos living on the Navajo Nation. The population is young with a median age of 28 years, and underemployed with a 55.9% reported unemployment rate, and an average household income of $27,389 (https://www.discovernavajo.com/fact-sheet.aspx). The NBCS was initiated to address Navajo community concerns about how chronic environmental exposure to uranium mine waste affects human health. The waste legacy from the Cold War mining includes a mixture of metals co-located geologically with the uranium and left behind, along with residual uranium, after extraction of the target uranium. The research team led by the University of New Mexico (UNM) Health Sciences Center Community Environmental Health Program included partnerships with Navajo Nation Department of Health, Navajo Area Indian Health Service (NAIHS), Southwest Research Information Center and the US Centers for Disease Control and Prevention Agency for Toxic Substances and Disease Registry (CDC/ATSDR) National Center for Environmental Health (NCEH). Written informed consent was obtained from all study participants and the study protocol approved by the University of New Mexico Institutional Review Board (HRPO 11–310) and the Navajo Nation Human Research Review Board (NNR 11.323).

In 2013, NBCS began recruiting pregnant women between 14 and 45 years of age who had lived on the Navajo Nation for at least 5 years, were willing to deliver at a participating NAIHS hospital, and have their child followed-up for 1 year postnatally. The NBCS has enrolled over six hundred pregnant women, and blood and urine samples were collected from each of the participant at the time of enrollment. The biomonitoring exposure data are comprised of metal exposures measured in three types of samples (blood, serum, and urine) from all NBCS participants when they enrolled. Metals were measured by the Centers for Disease Control and Prevention (CDC) Division of Laboratory Sciences. The biomonitoring metal exposure data have gone through the following strict quality control procedures: (1) The small proportion of duplicate analyses were averaged. Duplicates were highly concordant; (2) Urine measures were appropriately corrected for creatinine; (3) Concentrations for metals below the limit of detection (LOD) were imputed by LOD/√2; and (4) In order to reduce bias and data dimension, 11 metals with more than 40% missing values (due to below LOD or other non-LOD reasons) were excluded from analysis. The excluded metals include: Ethyl Mercury in Blood (BHGE), Methyl Mercury in Blood (BHGM), Inorganic Mercury in Blood (IHG), Arsenic (V) acid in Urine (UAS5), Arsenobetaine in Urine (UASB), Arsenocholine in Urine (UASC), Beryllium in Urine (UBE), Mercury in Urine (UHG), Monomethylarsinic Acid in Urine (UMMA), Platinum in Urine (UPT), Trimethylarsine in Urine (UTMO). These metals were excluded because their small variation will explain only a small proportion of the variation in the outcome variable, which may induce noise and limit the performance of statistical inference. The metal measurements were collected from the following matrices: blood (5 metals), serum (3 metals), and urine (16 metals). The list of metals analyzed in the NBCS along with their abbreviations is included in Table 1.

**Table 1:** List of metals with their abbreviations analyzed in the NBCS study

Abbreviations	Description of metal analytes in NBCS
BCD	Cadmium - Blood
BHGE	Ethyl Mercury - Blood
BHGM	Methyl Mercury - Blood
BMN	Manganese - Blood
BPB	Lead - Blood
BSE	Selenium - Blood
IHG	Inorganic Mercury - Blood
THG	Mercury Total - Blood
SCU	Copper - Serum
SSE	Selenium - Serum
SZN	Zinc - Serum
UAS3	Arsenous (III) acid - Urine
UAS5	Arsenic (V) acid - Urine
UASB	Arsenobetaine - Urine
UASC	Arsenocholine - Urine
UBA	Barium - Urine
UBE	Beryllium - Urine
UCD	Cadmium - Urine
UCO	Cobalt - Urine
UCS	Cesium - Urine
UDMA	Dimethylarsinic Acid - Urine
UHG	Mercury - Urine
UIO	Iodine - Urine
UMMA	Monomethylarsinic Acid - Urine
UMN	Manganese - Urine
UMO	Molybdenum - Urine
UPB	Lead - Urine
UPT	Platinum - Urine
USB	Antimony - Urine
USN	Tin - Urine
USR	Strontium - Urine
UTAS	Arsenic Total - Urine
UTL	Thallium - Urine
UTMO	Trimethylarsine - Urine
UTU	Tungsten - Urine
UUR	Uranium - Urine

The primary health outcome is the biomarker of oxidative stress, which is considered an important component of various diseases [30–35]. The oxidative stress outcome was measured by the ratio of 8-iso-PGF2α to prostaglandinF2α biomarkers, which has been established as a valid biomarker to distinguish enzymatic versus chemical lipid peroxidation [36, 37]. A subset of the maternal enrollment urine samples was selected for oxidative stress analysis, the basis of this investigation. The enrollment protocol prioritized recruitment of women during their 1st trimester but an open enrollment process was used, allowing women to enroll at any time during their pregnancy. We randomly selected 132 participants with 66 participants each from the two groups with serum zinc concentrations above and below the World Health Organization (WHO) level of sufficiency of 70 μg/dL.

In our recently published article which focused on hypothesis-driven restricted set of analysis [38], we have investigated the contributions of uranium, total arsenic, arsenous (III) acid (arsenite, AsIII), dimethylarsinic acid (DMA), and zinc to the oxidative stress biomarker outcome. For this investigation, we have broadened the exposure suite to include metals detected in more than 60% of the samples described as above. The characteristics of the study population were described in the Additional file 1: Table S4 of our previous publication [38] and are shown in Additional file 1: Table S1. In addition to the metals, we also included other demographic variables (age, ceremonial tobacco usage, employment, enrollment trimester and BMI) previously shown to be linked to the oxidative stress outcome [38]. The outcome variable oxidative stress biomarker is significantly different among ceremonial tobacco users vs non-users and among moms enrolled at different trimesters [38]. The correlations between the metals are described in Additional file 1: Figure S1. The maximum correlation is found to be 0.76. To conduct a fair comparison among metals that have different magnitudes of exposures, we first performed log transformation to reduce the skewness of the variable distributions, and then performed standardization using z score transformation in the statistical modeling. The distributions of the metals before and after standardization are described in boxplots (Additional file 1: Figure S2).

## Methods

### Overview

We propose to examine a two-step procedure of combining the random forest (RF) [25, 26] with adaptive lasso [27] approach for analyzing the chemical mixture data. We evaluated the performance of the proposed two-step method, using simulated datasets (dataset #1 provided by the NIEHS workshop, and datasets that we simulated to represent a larger number of correlated exposure variables with varying correlations) and a real-life dataset from the NBCS. We compared the proposed method with other approaches including (1) single step adaptive lasso; and (2) two-step with CART followed by the adaptive lasso approach which was reported previously [3] and was also widely utilized in the NIEHS workshop.

### Classification and regression trees (CART)

Classification and regression trees are machine-learning methods for constructing prediction models from data, obtained by recursively partitioning the data space and fitting a simple prediction model within each partition [39]. For continuous dependent variables, the prediction error for regression trees is estimated by the squared difference between the observed and predicted values, which can be used to select the variables with the most predictability.

### Random Forest (RF)

The Random Forest method is an ensemble-based approach which grows many trees. Each tree is built using a recursive portioning method to split the feature space, spanned by all predictor variables, into groups of subjects with similar association patterns between the predictor variables and the outcome variable. Specifically, each tree is grown using a randomly drawn bootstrap sample of the data. Based on a randomly selected subset of the variables, a criterion based on mean square error is used to split the tree nodes. Prediction is made by averaging over an ensemble of trees. We used the variable importance (VIMP) measure to pre-select important variables into the second stage analysis. VIMP is a measure of how important a variable is, which estimates the change in prediction error if that variable is eliminated from analysis. A larger VIMP value corresponds to better predictability of a variable.

### Adaptive lasso (alasso)

The lasso method proposed by Tibshirani [40] shrinks many coefficients towards zero by imposing an L_1_ norm penalty, which has been extensively studied in performing variable selection. Lasso estimates of unknown parameter Beta are defined as

1 $$ {\widehat{\boldsymbol{\beta}}}_{lasso}={argmin}_{\beta}\parallel \boldsymbol{y}\hbox{-} {\sum}_{j=1}^p{\boldsymbol{x}}_j{\beta}_j{\parallel}^2+\lambda {\sum}_{j=1}^p\left|{\beta}_j\right|, $$

Where ***y*** denotes the outcome variable, ***x***_*j*_, *j* = 1, … , *p* represents the *p* number of exposure variables, and *λ* is a nonnegative regularization parameter that can be determined by cross validation.

Although lasso is often successful, it has some limitations for certain situations. First, lasso produces biased estimates for the large coefficients. Second, in the existence of multiple highly correlated variables, lasso tends to arbitrarily pick only one or a few of them and shrinks the rest to zero. Adaptive lasso is a modified version of lasso that was developed by Zou [27] to address the limitations of lasso. Using a flexible weighting scheme, the adaptive lasso applies different amounts of shrinkages to different coefficients, whereas lasso applies the same penalty to every regression coefficient which may induce potential bias. Adaptive lasso combines the good features of both subset selection and ridge regression, which appears to perform well in the existence of multicollinearity.

Adaptive lasso penalizes the weighted L_1_-norm of the regression coefficients, and the coefficient estimates are defined as:

2 $$ {\widehat{\boldsymbol{\beta}}}_{alasso}={argmin}_{\boldsymbol{\beta}}\parallel \boldsymbol{y}\hbox{-} {\sum}_{j=1}^p{\boldsymbol{x}}_j{\beta}_j{\parallel}^2+\lambda {\sum}_{j=1}^p{w}_j\left|{\beta}_j\right|, $$

where ***w*** is a weighing vector with ${w}_j={\left|{\widehat{\beta}}_j^{initial}\right|}^{-\gamma }$. Zou [27] suggested a two-dimensional cross-validation to tune the adaptive lasso and find the optimal pair of (*γ*, *λ*), and suggested constructing the adaptive weights using initial ***β*** estimates by ${\widehat{\boldsymbol{\beta}}}_{(ols)}$ or ${\widehat{\boldsymbol{\beta}}}_{(ridge)}$ when multicollinearity is a concern. Their simulation results have demonstrated that the adaptive lasso compares favorably with other sparse modeling techniques.

### Performance evaluation

We compared the performance among three methods: (1) single step adaptive lasso; (2) two-step with CART followed by adaptive lasso approach; and (3) two-step with RF followed by adaptive lasso approach. For each of the methods, we fit two models with one including all the main effects for the exposure variables and another one including additionally all pairwise interaction terms between the exposure variables.

A number of performance metrics including R^2^, Adjusted R^2^, mean squared error (MSE), and mean squared prediction error (MSPE) were calculated to measure the performance of the three methods (each with or without the interaction terms) in analyzing the health effects of metal mixtures. We also performed 10-fold cross validation to calculate the prediction performance measures. Specifically, we randomly and equally divided the datasets into 10 parts. Then, for each iteration we used the remaining 9 parts as the training set to build a model, and evaluated the model prediction error on the remaining part. The prediction error of each partition was then combined to produce the 10-fold cross-validation estimates of prediction error.

MSPE is calculated as: ${\sum}_{k=1}^K\frac{n_k}{n}\mathrm{MSP}{\mathrm{E}}_k={\sum}_{k=1}^K\frac{n_k}{n}\ {\sum}_{i\in {C}_k}\frac{{\left({y}_i-{\widehat{y}}_i\right)}^2}{n_k}$, where *C*_1_, *C*_2_, … , *C*_*K*_ denote the K partitions of the data each with size of *n*_*k*_.

### Software

All statistical analyses and simulations were performed using statistical software R 3.4.2. Adaptive lasso models were fit using the glmnet package, and Random Forest model selection was performed using the randomForestSRC package within the R.

## Results

### Simulation data set 1: NIEHS workshop dataset

We applied the proposed method to the simulated dataset #1 provided by the NIEHS workshop (https://www.niehs.nih.gov/about/events/pastmtg/2015/statistical/). These synthetic data can be viewed as the results of a prospective epidemiologic cohort study, which consists of correlated exposure variables *X* = (*X*_1_, *X*_2_, … , *X*_7_), a potential confounder variable (Z), and a continuous outcome variable (Y) resulting from a biologically-based dose response function of the exposure variables, plus the effects of confounding variables and normally distributed random noise. The simulation was set such that exposures *X*_1_ and *X*_2_ contributed positively to the outcome with *X*_1_ twice as potent as *X*_2_; *X*_4_ and *X*_5_ contributed negatively to the outcome with *X*_5_ 4.5 times as potent as *X*_4_; and *X*_7_ contributed positively to the outcome. *N* = 500 samples were included in the synthetic dataset.

**Fig. 1:**
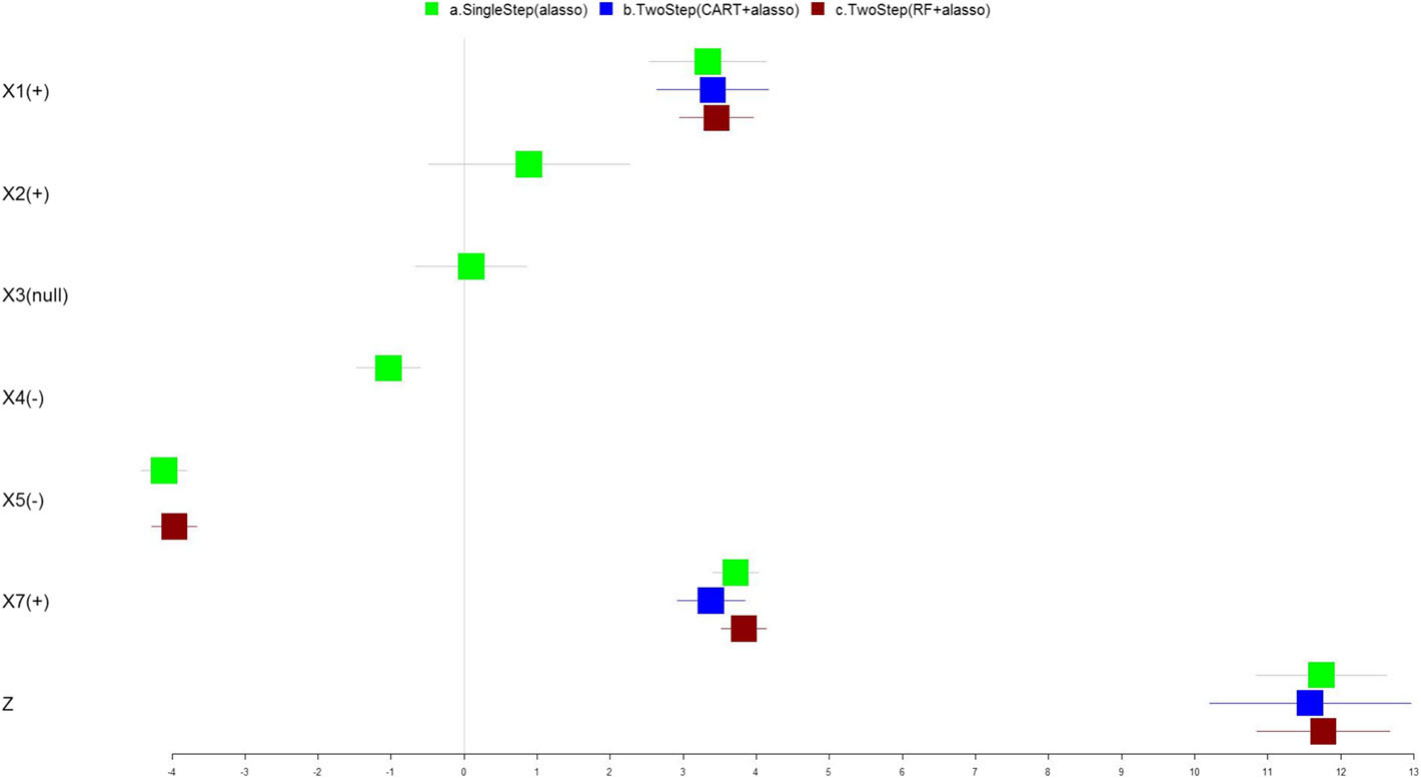
Simulated Data from the NIEHS Workshop (No Interaction). The forest plot describing the regression coefficients (boxes) and their 95% confidence intervals (lines) for modeling the relationship between exposure variables and outcome using three methods: (a) single step adaptive lasso (green); (b) two-step with CART followed by adaptive lasso approach (blue); and (c) two-step with RF followed by adaptive lasso approach (red). The numbers on X axis represent the magnitude of the regression coefficients. True nonzero effects were labeled on for variables on the Y axis in parentheses

The selected covariates and model performance of the three methods, modeling the main effects of exposure variables are shown in Fig. 1, Table 2, and Additional file 1: Table S2. Figure 1 is a forest plot describing the regression coefficients modeling the relationship between exposure variables and outcome (boxes) and their 95% confidence intervals (CI) (lines) using the three methods which are color coded as indicated in the legends. The single step adaptive lasso method identified a positive association between *X*_1_ and *X*_7_ and the outcome, and a negative association between *X*_4_ and *X*_5_ with the outcome with *X*_5_ 4.1 times as potent as *X*_4_. Two variables, *X*_2_ and *X*_3_, are included in the final selected model, but the coefficients are not statistically significantly different from zero. The RF followed by adaptive lasso method identified *X*_1_, *X*_5_ and *X*_7_ to be significantly associated with the outcome, where the coefficient estimates are similar to those identified using the single step adaptive lasso approach. While *X*_2_ and *X*_4_ which have the smaller magnitude of association effects were not identified, the total variance explained by the selected model is close to the more complex model by the single step adaptive lasso method (adjusted R2 are 91.9 and 92.2%, respectively). The MSPE are about the same (9.47 vs. 9.55). The CART followed by adaptive lasso method properly identified the association of *X*_1_ and *X*_7_, which accounts for only 81% of the total outcome variance. Adjusted R2 is about 10% less compared to other two methods, and the MSPE is much larger.

**Table 2:** Performance Evaluation for Each Approach for NIEHS Dataset ^a^ (No Interaction)

	Adaptive lasso	CART+ Adaptive lasso	RF+ Adaptive lasso
R^2^	92.3%	81.5%	91.9%
Adjusted R^2^	92.2%	81.4%	91.9%
MSE	9.080	21.73	9.50
MSE.CV	9.082	21.72	9.076
MSPE.CV	9.47	22.00	9.55

^a^The dataset includes 500 samples and 8 predictor variables with Pearson’s correlations ranging from −0.13 to 0.89. *MSE* Mean square error, MSE. *CV* Cross validation, *MSPE* Mean square predictive error

**Fig. 2:**
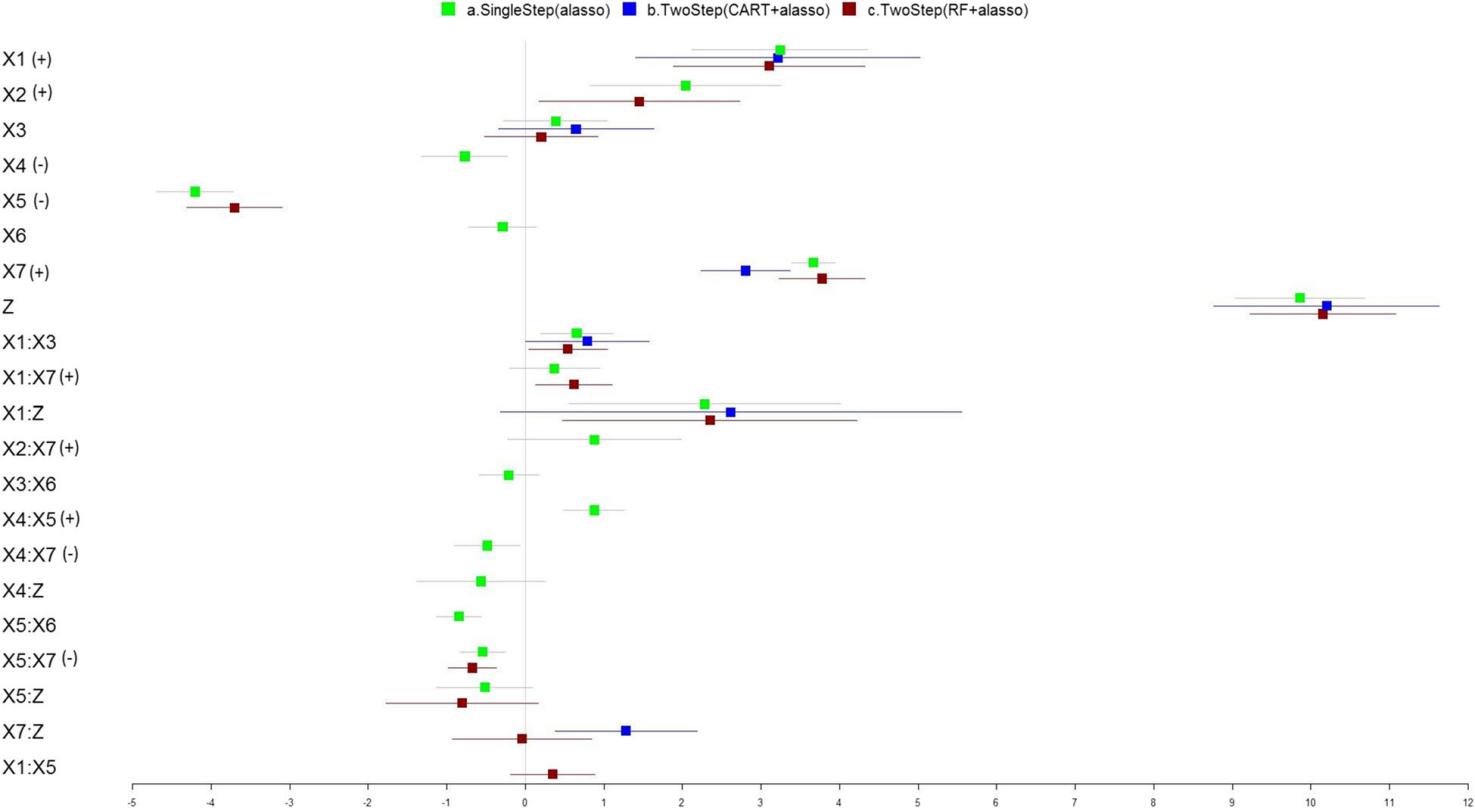
Simulated Data from the NIEHS Workshop (Including Pairwise Interaction Terms). The forest plot describing the regression coefficients (boxes) and their 95% confidence intervals (lines) for modeling the relationship between exposure variables and outcome using three methods: (a) single step adaptive lasso (green); (b) two-step with CART followed by adaptive lasso approach (blue); and (c) two-step with RF followed by adaptive lasso approach (red). The numbers on X axis represent the magnitude of the regression coefficients. True nonzero effects were labeled for variables on the Y axis in parentheses

The selected covariates and model performance of the three methods, modeling the main and pairwise interaction effects of exposure variables, are shown in Fig. 2, Table 3, and Additional file 1: Table S3. The single step adaptive lasso method properly identified the synergistic interaction between *X*_4_ and *X*_5_, and the antagonistic interactions between *X*_4_ and *X*_7_, and *X*_5_ and *X*_7_. Adjusted R2 was increased slightly from 92.2 to 94.5%. The two-step RF followed by the adaptive lasso method properly identified a different interaction: the synergistic interaction between *X*_1_ and *X*_7_. The adjusted R2 was increased from 91.9 to 93.4%, which is slightly smaller than that using the single-step adaptive lasso method. Similarly, the MPSE for the models including the interaction terms decreased for both the single-step adaptive lasso and two-step RF + adaptive lasso method, where the two-step approach yields a slightly higher MSPE. One explanation is that the relatively small effect of *X*_4_ on the outcome was not preselected to be an important predictor variable using the RF method, therefore interaction terms between *X*_4_ and other variables were not detected. The two-step CART followed by adaptive lasso method failed to identify any of the pre-specified interaction effects in the simulation setting, and the adjusted R2 for the model including interaction terms improved by a few percentage while the MSPE remained high.

**Table 3:** Performance Evaluation for Each Approach for NIEHS Dataset ^a^ (Including Interaction Terms)

	Adaptive lasso	CART+ Adaptive lasso	RF+ Adaptive lasso
R^2^	94.7%	83.4%	93.6%
Adjusted R^2^	94.5%	83.1%	93.4%
MSE	6.20	19.56	7.56
MSE.CV	6.10	19.34	7.50
MSPE.CV	7.11	20.76	8.14

^a^ The dataset includes 500 samples and 8 predictor variables with Pearson’s correlations ranging from − 0.13 to 0.89. The pairwise interaction terms among the predictor variables were included in the modeling

The major findings from the application to the simulation dataset by NIEHS are that while the two-step approach identified less presumed association effects and interaction effects, it discovered a more parsimonious model which had a comparable performance compared to the single step approach. While RF is a tool that can effectively identify important variables and perform data dimension reduction, it may not help much in analysis of small dimension datasets such as the one provided in the workshop.

### Simulation data set 2: correlated dataset with variables that mimic real-life exposure profile

**Fig. 3:**
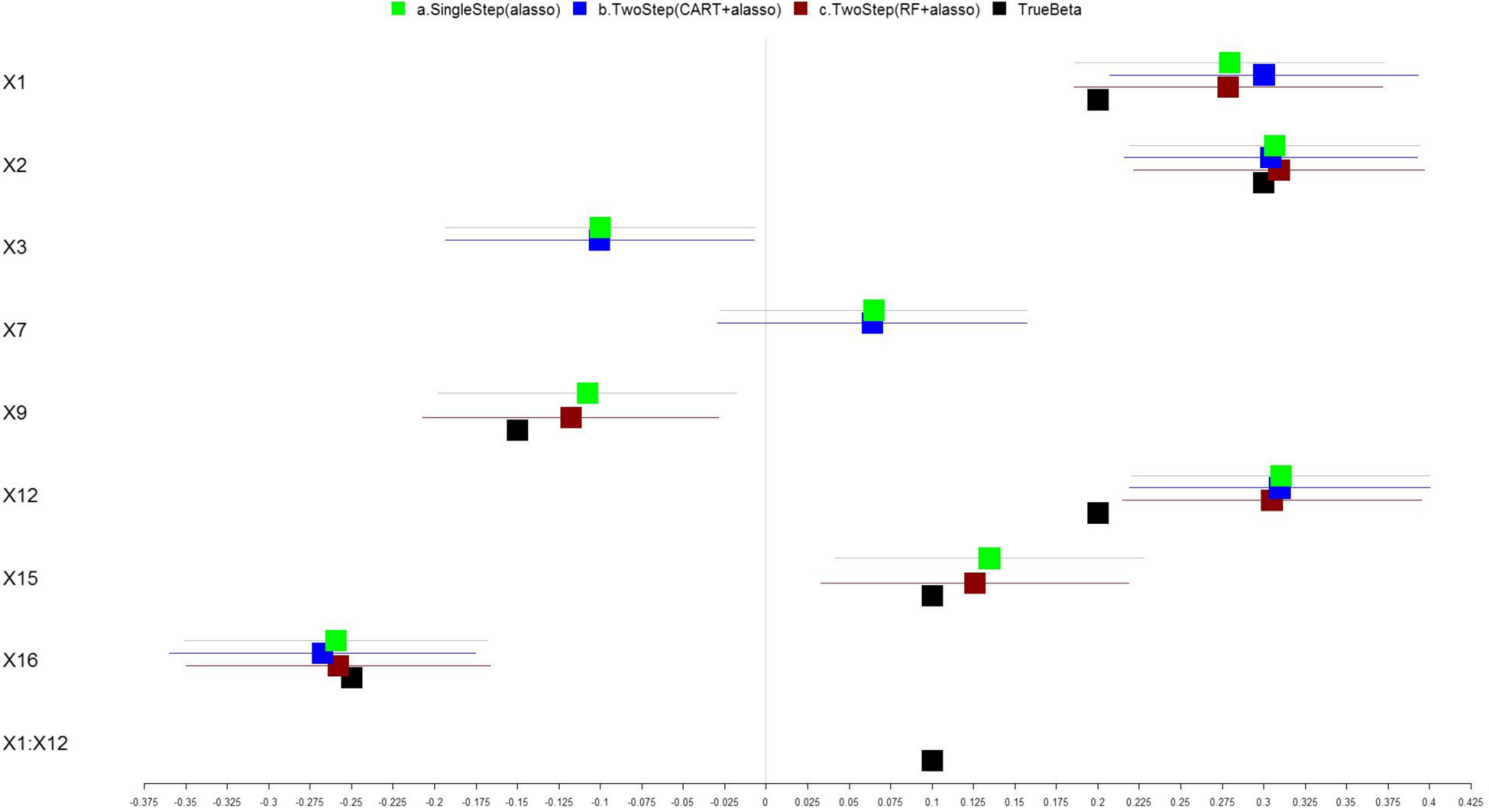
Analysis of Local Simulated Dataset (No Interaction). The forest plot describing the regression coefficients (boxes) and their 95% confidence intervals (lines) for modeling the relationship between exposure variables and outcome using three methods: (a) single step adaptive lasso (green); (b) two-step with CART followed by adaptive lasso approach (blue); and (c) two-step with RF followed by adaptive lasso approach (red). The black boxes indicate the true parameter coefficients used in the simulation. True nonzero covariates were set to include: main effects of X1, X2, X9, X12, X15, X16, and interaction effects between X1 and X12. The numbers on X axis represent the magnitude of the regression coefficients

**Fig. 4:**
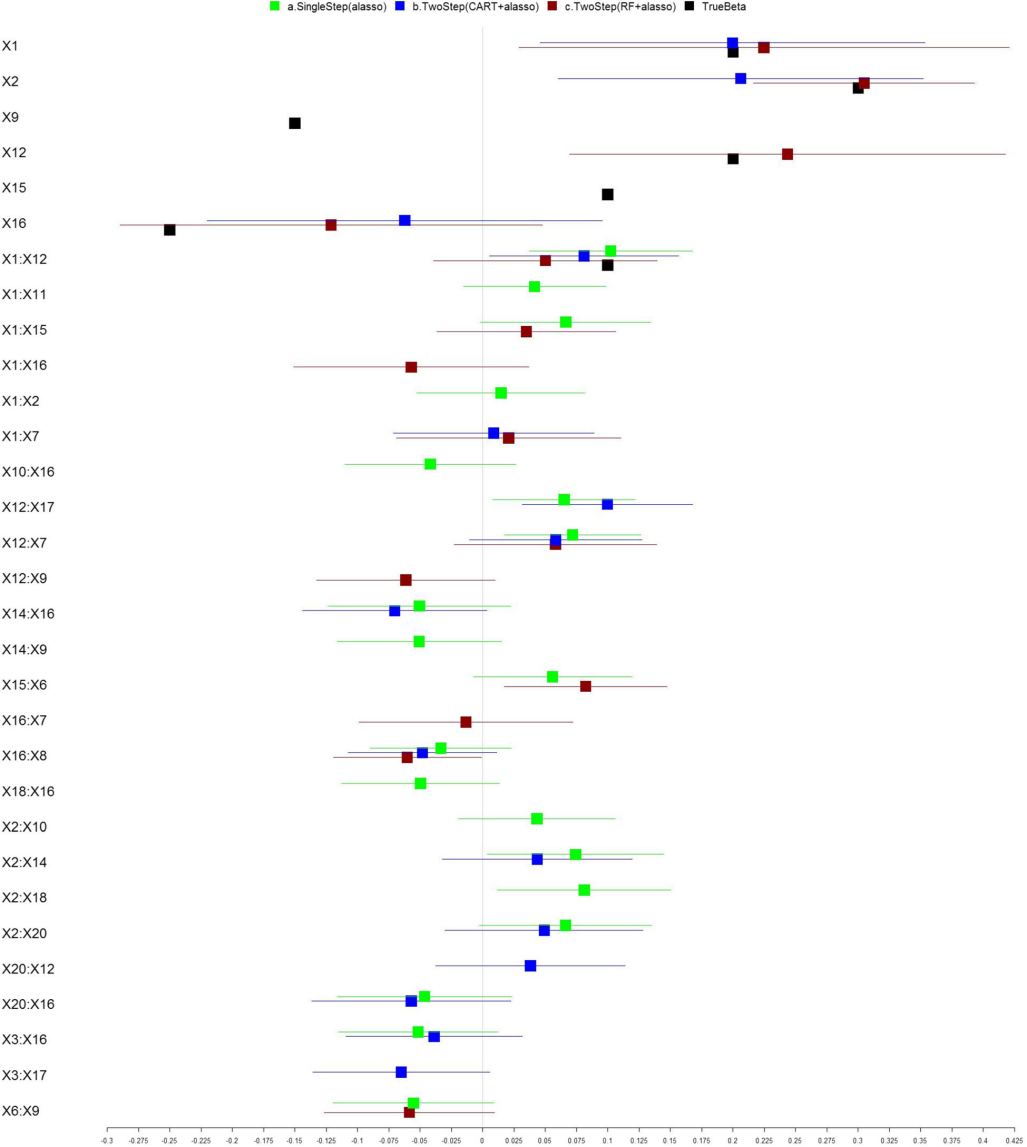
Analysis of Local Simulated Dataset (With Interaction). The forest plot describing the regression coefficients (boxes) and their 95% confidence intervals (lines) for modeling the relationship between exposure variables and outcome using three methods: (a) single step adaptive lasso (green); (b) two-step with CART followed by adaptive lasso approach (blue); and (c) two-step with RF followed by adaptive lasso approach (red). The black boxes indicate the true parameter coefficients ($\widehat{\beta}$) used in the simulation. True nonzero covariates were set to include: main effects of X1, X2, X9, X12, X15, X16, and interaction effects between X1 and X12. The numbers on X axis represent the magnitude of the regression coefficients

We simulated another dataset which consists of a larger number (*m* =20) of correlated exposure variables *X* = (*X*_1_, *X*_2_, … , *X*_20_), and a continuous outcome variable (Y) resulting from the linear combination of the main effects and interaction effects of a subset of the exposure variables, plus normally distributed random noise. *N* = 500 samples were simulated. The simulation was set to include the positive association effects of exposures *X*_1_, *X*_2_, *X*_12_ and *X*_15_, negative effects of *X*_9_ and *X*_16_, as well as a synergistic interaction between *X*_1_ and *X*_12_. We assume a correlation of *r* = 0.1 among variables (*X*_1_, *X*_2_,  … , *X*_15_) and a correlation of *r* =0.05 among variables (*X*_16_, *X*_17_,  … , *X*_20_) . The true model coefficients and those identified using the three methods with the main effects model are shown in Fig. 3 and Additional file 1: Table S4, their model performance measures are shown in Table 4. The corresponding results of models including pairwise interaction effects are shown in Fig. 4 and Table 5, and Additional file 1: Table S5. The two-step RF + Adaptive lasso method correctly identified the main effects of the non-zero coefficients and shrunk the rest to zero. While the single-step adaptive lasso method detected the true association effects for (*X*_1_, *X*_2_, *X*_9_, *X*_12_, *X*_15_, and *X*_16_), it also falsely detected null effects for *X*_3_ and *X*_7_. The two-step CART+adaptive lasso method falsely detected null effects for *X*_3_ and *X*_7_, and also failed to detect the true effects of *X*_9_ and *X*_15_, yielding poorer performance (small adjusted R^2^ and larger MSPE). The performance for single-step adaptive lasso and two-step RF + adaptive lasso is similar as measured by comparable adjusted R^2^ and smaller MSPE using cross validation. Again, the two-step RF + adaptive lasso approach yielded a parsimonious model with less complexity. Adding pairwise interaction terms to the two-step RF + adaptive lasso method in addition detected the interaction effects between *X*_1_ and *X*_12_. While several null interaction effects were falsely detected in all three models which may result from the complex correlation structure among the multiple exposure variables, the false detection rate is the lowest for our proposed two-step approach.

**Table 4:** Performance Evaluation for Each Approach for Local Simulated Dataset ^a^ (No Interaction)

	Adaptive lasso	CART+ Adaptive lasso	RF+ Adaptive lasso
R^2^	30.0%	28.2%	29.2%
Adjusted R^2^	28.9%	27.3%	28.3%
MSE	1.012	1.039	1.025
MSE.CV	1.003	1.038	1.017
MSPE.CV	1.095	1.088	1.078

^a^ The dataset includes 500 samples and 20 predictor variables with Pearson’s correlations ranging from − 0.13 to 0.22

**Table 5:** Performance Evaluation of Each Approach for Analysis of Local Simulated Dataset ^a^ (With Interaction)

	Adaptive lasso	CART+ Adaptive lasso	RF+ Adaptive lasso
R^2^	33.6%	31.6%	32.6%
Adjusted R^2^	31.0%	29.4%	30.7%
MSE	0.961	0.990	0.975
MSE.CV	0.926	0.990	0.967
MSPE.CV	1.206	1.096	1.099

^a^The dataset includes 500 samples and 20 predictor variables with Pearson’s correlations ranging from − 0.13 to 0.22. The pairwise interaction terms among the predictor variables were included in the modeling

To determine the model performance with varying degrees of correlations among predictor variables, we have performed simulation with various increasing correlations among predictor variables (*r* =0.3, 0.5, 0.7, and 0.8), where same number of variables (*m* = 20) and samples (*N* = 500) were simulated as previously described in the simulation data set 2. For each of the correlation settings, we have compared the estimated regression coefficients with the true simulation coefficients and provided model performance comparison tables for the three methods: single step adaptive lasso, CART+adaptive lasso, and RF + adaptive lasso, each modeling with or without interaction effects. These results were summarized in Additional file 1: Figures S3, S4 and Tables S6, S7 for *r* =0.3; Additional file 1: Figures S5, S6 and Tables S8, S9 for *r* =0.5; Additional file 1: Figures S7, S8 and Tables S10, S11 for r = 0.7; and Additional file 1: Figures S9, S10 and Tables S12, S13 for r = 0.8. The results from these additional simulations with increasing correlations among the predictor variables suggest the following: single step adaptive lasso approaches tend to estimate a model that includes the true model variables as a subset [41], i.e. it identifies more variables than the true model. The RF approach in the first step performs effective screening and dimension reduction that retains a subset of variables with either linear or nonlinear relationships with the outcome of interest. Our proposed two-step approach is more likely to detect a parsimonious or sparse model that is closer to the true model, which in the meantime have prediction performances better than or as well as the single step approaches. Improved results are consistently observed for simulated datasets with modest to moderately large correlated predictor variables (r < 0.8). However, when the correlations are extremely large (e.g. r > 0.8), none of the three approaches performs very well, with each falsely including a subset of true null covariates into the final model. In such cases, pre-filtering the data prior to analysis by removing one variable from the highly correlated pairs based on biological inputs may help improve the model performance. This is worthy for further investigation, but it is beyond the current research.

### Application to the NBCS

**Fig. 5:**
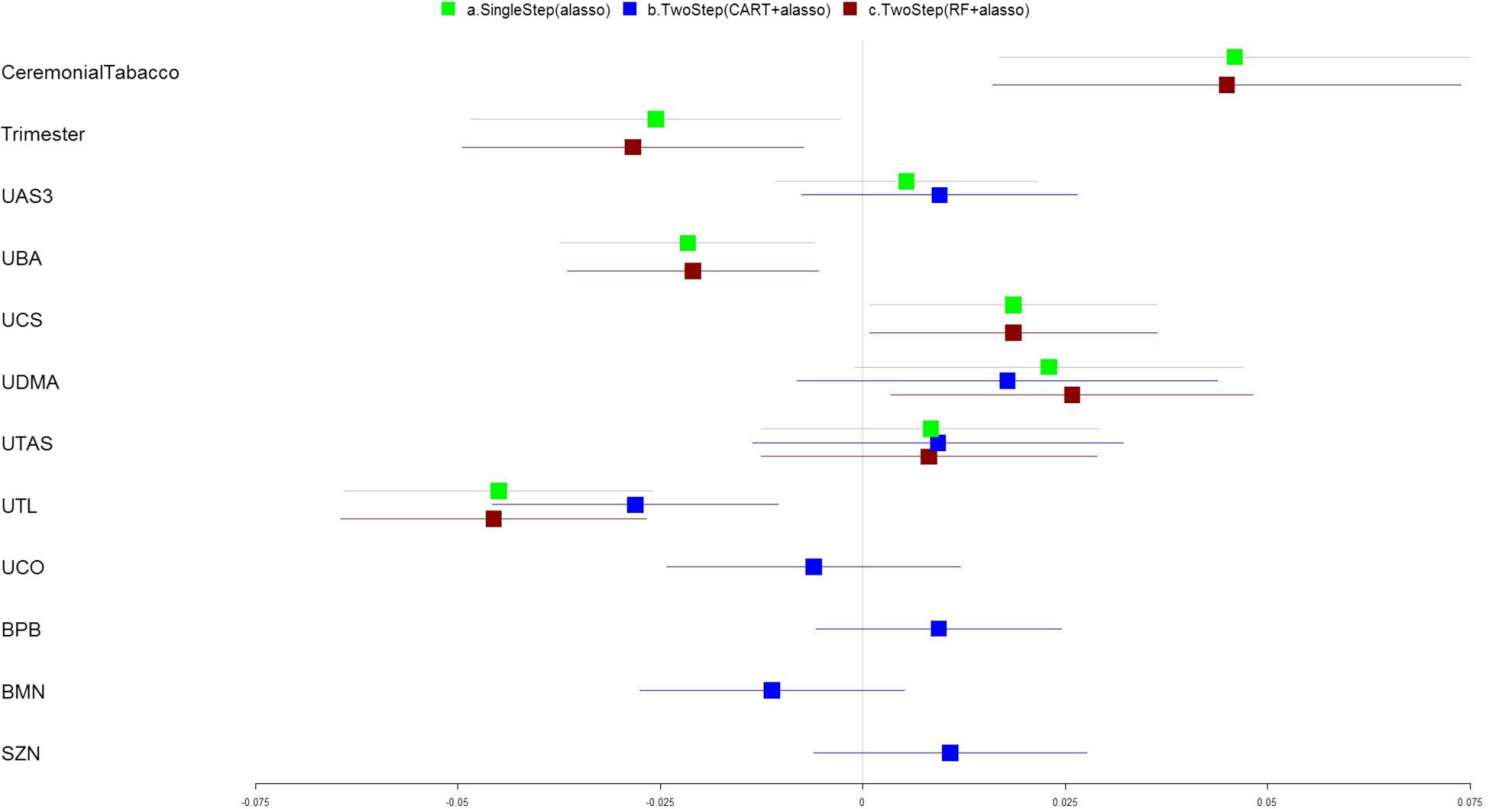
Association analyses of the metal exposures and oxidative stress biomarkers collected in the NBCS (No Interaction). The forest plot describing the regression coefficients (boxes) and their 95% confidence intervals (lines) for modeling the relationship between exposure variables and outcome using three methods: (a) single step adaptive lasso (green); (b) two-step with CART followed by adaptive lasso approach (blue); and (c) two-step with RF followed by adaptive lasso approach (red). The numbers on X axis represent the magnitude of the regression coefficients

**Fig. 6:**
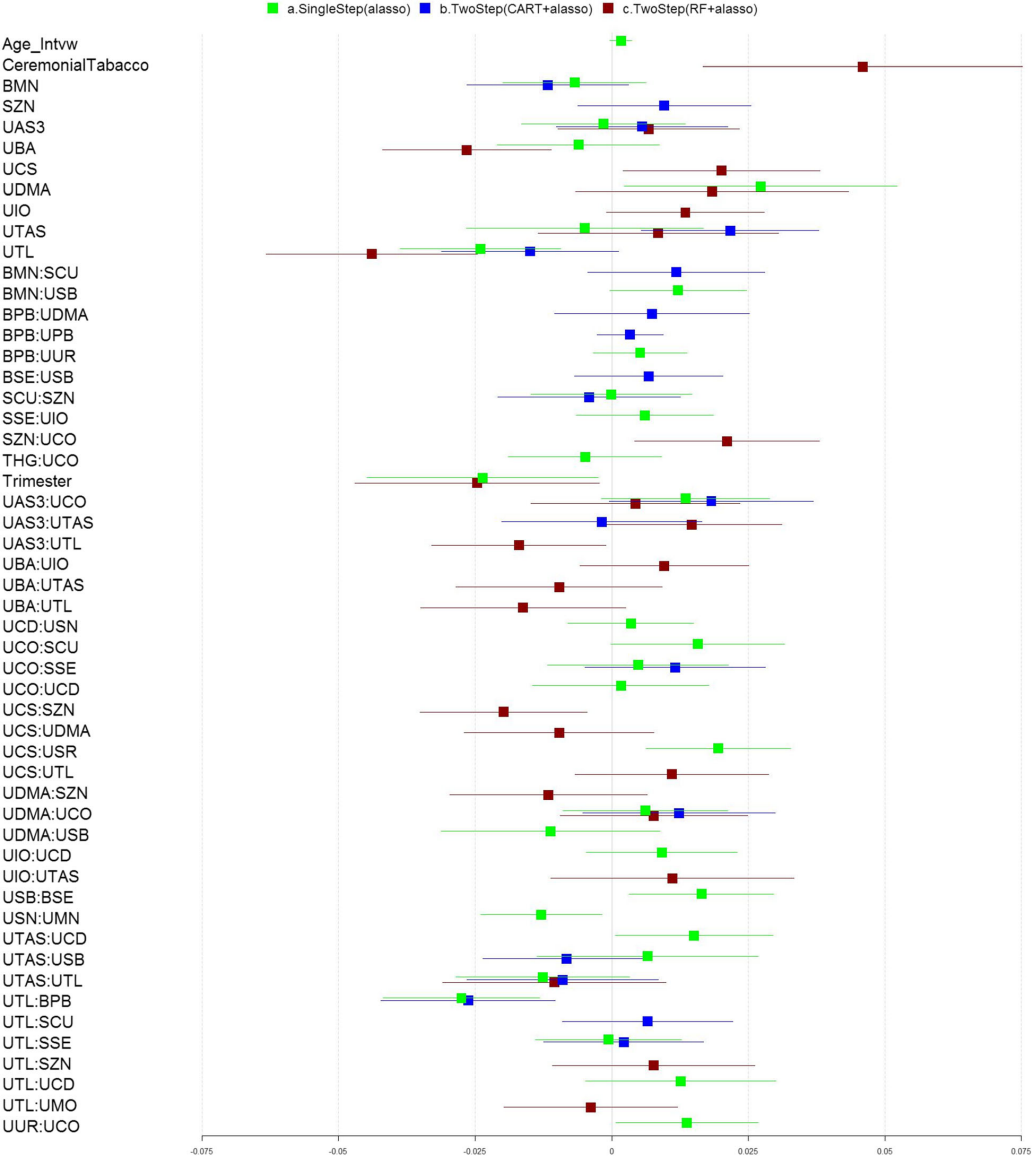
Association analyses of the metal exposures and oxidative stress biomarkers collected in the NBCS (With Interaction). The forest plot describing the regression coefficients (boxes) and their 95% confidence intervals (lines) for modeling the relationship between exposure variables and outcome using three methods: (a) single step adaptive lasso (green); (b) two-step with CART followed by adaptive lasso approach (blue); and (c) two-step with RF followed by adaptive lasso approach (red). The numbers on X axis represent the magnitude of the regression coefficients

The comparison results of applying the three methods to analysis of the effects of the metal mixture exposures on the oxidative stress biomarker collected in the NBCS are shown in Fig. 5, Table 6, and Additional file 1: Table S14 (without interaction), and Fig. 6, Table 7, and Additional file 1: Table S15 (with interaction). Potential confounding variables such as smoking, pregnancy trimester, and age at interview were evaluated and appropriately adjusted for in the model. The proposed two-step RF + adaptive lasso method yielded a model with the largest adjusted R2 and smaller MSPE using cross validation for the main effects modeling (Table 6). We observed positive associations between oxidative stress biomarkers and exposures to cesium and dimethylarsinic acid in urine, and negative associations for exposures to barium and thallium in urine (Fig. 5). For the models including pairwise interaction terms, the proposed two-step RF + adaptive lasso method detected the main effects of barium, cesium, and thallium in urine, interactions between zinc in serum and cobalt in urine, between zinc in serum and cesium in urine, and between arsenous (III) acid and thallium in urine in relation to the oxidative stress outcome. Our proposed two-step method yielded the smallest MSPE. The single-step adaptive lasso yielded a model with largest adjusted R2 and included more interaction effects terms than other models. However, this may illustrate a possible overfitting which has caused the larger MSPE using cross validation. In this application to the NBCS dataset, the two-step CART + adaptive lasso method also had poorer performance (smaller adjusted R2 and larger MSPE) compared to the other two methods.

**Table 6:** Performance Evaluation of each approach used in analyses of NBCS ^a^ (No Interaction)

	Adaptive lasso	CART+ Adaptive lasso	RF+ Adaptive lasso
R^2^	37.0%	21.5%	36.7%
Adjusted R^2^	32.2%	15.5%	32.6%
MSE	0.0048	0.0061	0.0049
MSE.CV	0.0047	0.0061	0.0047
MSPE.CV	0.0078	0.0078	0.0062

^a^The dataset includes 132 samples and 30 predictor variables including 25 metals and 5 demographic variables (age, smoking, employment, trimester and BMI). The Pearson’s correlations between the metals range from −0.32 to 0.76

**Table 7:** Performance Evaluation of each approach used in analyses of NBCS^b^ (With Interaction)

	Adaptive lasso	CART+ Adaptive lasso	RF+ Adaptive lasso
R^2^	65.5%	43.4%	56.01%
Adjusted R^2^	52. 6%	32.1%	43.7%
MSE	0.0027	0.0044	0.0034
MSE.CV	0.0023	0.0041	0.0035
MSPE.CV	0.0107	0.011	0.0088

^b^ The dataset includes 132 samples and 30 predictor variables including 25 metals and 5 demographic variables (age, smoking, employment, trimester and BMI). The Pearson’s correlations between the metals range from − 0.32 to 0.76. The pairwise interaction terms among the metal exposure variables were included in the modeling

## Discussion

We proposed to use a two-step approach combining the Random Forest (RF) and adaptive lasso methods to analyze the relationship between mixtures of metal exposures and health outcomes which incorporates detection of the interaction effects in addition to main effects. This work aims to improve the model performance of a two-step with CART and lasso approach previously reported by others [3]. The CART method is a single tree method, which has some known limitations. First, trees tend to be non-robust, i.e. a small change in the training dataset can result in a big change in the tree and its predictions. Second, the CART algorithm yields locally optimal trees which does not guarantee globally optimal trees. Third, CART may suffer from the overfitting problem by generating an overly-complex tree which does not generalize well to independent test data.

The RF method utilized in the first step provides more robust results than CART because of the two randomnesses included in the algorithm (bootstrapped samples for training each tree and a randomly chosen subset of candidates for splitting candidates), and use of “out-of-bag” predictions to evaluate model performance. After the RF is applied to reduce the data dimension, we utilize the adaptive lasso approach in the second step to perform additional variable selection and shrinkage. It was reported previously that standard lasso approaches tend to estimate a model that includes the true model variables as a subset [41], and often times the selected model covariates are not stable due to correlations among a large number of predictor variables. We have observed similar results in our simulations, i.e., the lasso identifies more variables than the true model. Our two-step approach utilizes RF approach in the first step to select variables associated with the outcome that have either linear or nonlinear relationships. This step aims to largely reduces the data dimensionality especially when interaction effects are necessary to be considered in the model, as illustrated in our motivating data. After the pre-filtering of variables in the first step, the adaptive lasso approach in the second step is more likely to identify a parsimonious or sparse model that has prediction performances better than or as well as the single step approaches. Our two-step approach provides a flexible weighted penalty to the coefficients and overcomes the limitations of lasso in generating biased estimates for large coefficients or when multicollinearity among variables exist. This proposed two-step approach provides an effective way of analyzing metal-mixture exposures and their impact on health outcomes by combining variable selection and adaptive shrinkage strategies.

Using hypothetical datasets and an application to a real-life dataset from the Navajo Birth Cohort Study, we observed that the proposed two-step method performs effective variable dimension reduction and identifies a parsimonious model that outperforms or performs as well as the other comparison methods (single-step approach and two-step CART + adaptive lasso) by maintaining comparable adjusted R^2^ and mean squared prediction errors from cross validation.

There are some limitations of this modeling approach. While the RF method provides the utility to select important variables, which accounts for the nonlinear relationship between exposure and outcome variables, the final model as determined in the second step using the adaptive lasso approach only captures the linear relationship between the outcome and exposure variables (independent metals or interacting metals). In the cases when excessive correlations exist among a group of predictor variables, lasso approaches may only select one variable from that group and provide biased estimates of the effects. We will explore an alternative elastic net [42] approach in our future work, which is a penalized regression approach using a combination of lasso [40] and ridge [43] penalty and is expected to perform variable selection and handle correlated predictors at the same time. The elastic net approach overcomes the limitation of lasso by encouraging grouping effects, which tend to select groups of highly correlated variables into the model. How to model the complex nonlinear relationships between exposure and outcome variables or the joint effects of multiple exposures on the outcome remains a big analytic challenge. While all three methods we compared provide some power in detecting the true interaction effects, they also falsely detected some null interaction effects probably due to the large number of interaction effect terms or the complex correlation patterns among multiple exposure variables. Statistical methods with improved power as well as the ability to control false discoveries need further development. We acknowledge that examining the model performances using specific simulated datasets have some limitations as compared to running a simulation study which repeats simulations thousands of times under multiple different scenarios. In this investigation, we focused on using simulation datasets and real NBCS dataset to evaluate the performance of our proposed approach, and to provide general recommendations of our approach along with its strengths and limitations. We will explore more comprehensive simulation studies in future work. This exploratory analysis evaluating the relationship between the mixture metal exposures and oxidative biomarker analysis is consistent with our previous recently published article in this population [38], but expands those findings. That initial evaluation which focused on hypothesis-driven analyses of uranium, arsenic, and zinc, we have found a positive association between Arsenic and increased levels of an oxidative stress biomarker and no evidence of association between Uranium and the oxidative stress biomarker. Zinc was not found to be directly related to the oxidative stress biomarker, but modified the relationship between arsenic and the oxidative stress biomarker [38], which is similar to our current findings. Additionally, we have discovered the associations between oxidative stress biomarkers and other metals exposures (cesium, barium, dimethylarsinic acid, thallium, interaction between zinc and cobalt, interaction between zinc and cesium). Some of the findings are supported by previous literature while others are not directly mentioned or implicated in literature and warrant further investigation. Components of our findings on the associations between oxidative stress biomarkers and exposures to cesium, dimethylarsinic acid, barium, and thallium in the NBCS are supported by previous literature reports [44–47] in human and animal models. There is experimental evidence that oxidative stress can be induced either by redox-cycling metals such as barium or thallium through directly generating free radicals, or indirectly by non-redox cycling metals such as arsenic, lead or mercury [44, 45]. A recent study in the general Spanish population has reported an association between elevated oxidative stress and barium [48]. Hanzel et al. 2005 reported a link between thallium and oxidative stress through glutathione (GSH) metabolism and peroxide detoxification [46]. A recent study on plants reported that cesium exposure reduced plant growth via activating the defense mechanism against oxidative stress [47]. In a previous mouse model, cesium induced renal and liver damage through oxidative stress [49]. We also identified the interaction effects between zinc and cobalt and between zinc and cesium, which are implicated by previous experimental studies and require further validation. It is surprising that we identified positive associations with oxidative stress for dimethylarsinic acid and cesium, but opposite negative associations for barium and thallium. These previous reports did not consider the complexity of exposures examined in the NBCS, but the consistency of their findings with our results underscore the complexity of the exposure-response relationship between metal-mixture exposures and this important biological pathway. Considering the limited research conducted in human populations, these results suggest that future research should be conducted in order to clearly understand the role of metal-mixture exposures in oxidative stress, the DNA damage process, and complex biological mechanisms underlying the adverse human health.

## Conclusions

There is no consensus as to how to appropriately analyze the health effects of metal mixture exposures, or recommendations on selection of approaches that best characterize study data. In an effort to address this challenge we proposed to use a two-step approach combining the strengths of random forest and adaptive lasso methods which incorporated detection of interaction effects in addition to main effects. This method was demonstrated to have favorable performance overall and extensive improvement in the ability to identify complex interaction effects between exposures using simulation example datasets and an application to data from the Navajo Birth Cohort Study. In summary, the major findings and recommendations of using our approach include the following:

### The RF + adaptive lasso two-step approach adds the following to existing methodology

- The RF screening step performs effective dimension reduction that retains variables with either linear or nonlinear relationships with the outcome of interest
- Overcomes the limitation that standard lasso approaches identify unstable models with multiple interaction terms which yield similar prediction utility
- Addresses the overfitting problem where standard approaches tend to estimate a model that includes more covariates than just the true nonzero covariates
- Provides a parsimonious or sparse model that is closer to the true model which in the meantime has prediction performances better than or as well as the single step approaches

### This two-step approach is appropriate under the following conditions

- When datasets consist of 10–50 predictor variables
- When estimation of the interaction effects and quantification of the effect sizes are of interest
- When modest to moderately large correlations (*r* < 0.8) exist among the predictor variables

### Limitations that still need to be addressed

- No approach performs well when extremely large correlations among predictor variables exist, and it is recommended to remove one variable from the highly correlated pairs based on biological inputs prior to analysis
- The identified interaction effects reflect statistical interactions only, and proper interpretation of the biological or toxicological impact requires content expertise
- There is increasing interest in detection of nonlinear and nonadditive relationships between metal mixtures and health outcomes or biomarkers, but there are relatively few statistical methods available. The Bayesian kernel machine regression (BKMR) is one approach to estimate the joint health effects of mixture exposures that can accommodate nonlinear relationships [50].
- There are limited statistical methods for analyses of chemical mixture data from more complex designs (e.g. time series or longitudinal data). The BKMR approach [50] can handle repeated measures, and the more recently developed Bayesian varying coefficient kernel machine regression (BVCKMR) is designed to estimate the mixture effects on outcome trajectories [51]. These two approaches characterize the exposure-response surface and provide visualizations of the interaction effects between mixture components for longitudinal data. Quantification of the evidence of interaction effects among mixtures on the outcome trajectories remains an analytical challenge.

## Additional file

Additional file 1: **Table S1.** Summary statistics for oxidative stress prostaglandin ratio biomarker by demographic characteristics. **Figure S1.** Correlation matrix among metals in the NBCS dataset. The pairwise correlation between the metals as measured by the Pearson’s r between log transformed metal exposures are shown in the figure below, color coded by the magnitude of the correlation. **Figure S2.** Distribution of the metals in the NBCS dataset before and after standardization are described and compared using boxplots. **Table S2.** Simulated Data from the NIEHS Workshop (No Interaction). **Table S3.** Simulated Data from the NIEHS Workshop (Including Pairwise Interaction Terms). **Table S4.** Analysis of Local Simulated Dataset 2 (No Interaction). **Table S5.** Analysis of Local Simulated Dataset 2 (With Interaction). **Figure S3.** Analysis of Local Simulated Dataset (No Interaction, rho=0.3). **Table S6.** Performance Evaluation for Each Approach for Simulated Dataset (No Interaction, rho=0.3). **Figure S4.** Analysis of Local Simulated Dataset (With Interaction, rho=0.3). **Table S7.** Performance Evaluation of Each Approach for Analysis of Local Simulated Dataset (With Interaction, rho=0.3). **Figure S5.** Analysis of Local Simulated Dataset (No Interaction, rho=0.5). **Table S8.** Performance Evaluation for Each Approach for Simulated Dataset (No Interaction, rho=0.5). **Figure S6.** Analysis of Local Simulated Dataset (With Interaction, rho=0.5). **Table S9.** Performance Evaluation of Each Approach for Analysis of Local Simulated Dataset (With Interaction, rho=0.5). **Figure S7.** Analysis of Local Simulated Dataset (No Interaction, rho=0.7). **Table S10.** Performance Evaluation for Each Approach for Simulated Dataset (No Interaction, rho=0.7). **Figure S8.** Analysis of Local Simulated Dataset (With Interaction, rho=0.7). **Table S11.** Performance Evaluation of Each Approach for Analysis of Local Simulated Dataset (With Interaction, rho=0.7). **Figure S9.** Analysis of Local Simulated Dataset (No Interaction, rho=0.8). **Table S12.** Performance Evaluation for Each Approach for Simulated Dataset (No Interaction, rho=0.8). **Figure S10.** Analysis of Local Simulated Dataset (With Interaction, rho=0.8). **Table S13.** Performance Evaluation of Each Approach for Analysis of Local Simulated Dataset (With Interaction, rho=0.8). **Table S14.** Association analyses of the metal exposures and oxidative stress biomarkers collected in the NBCS study (No Interaction). **Table S15.** Association analyses of the metal exposures and oxidative stress biomarkers collected in the NBCS study (With Interaction). (DOCX 4536 kb)

## Abbreviations

CART: Classification and regression trees
CV: Cross validation
Lasso: Least absolute shrinkage and selection operator
MSE: Mean squared error
MSPE: Mean squared prediction error
RF: Random forest
