# Supplementary material for: Two-step approach for assessing the health effects of environmental chemical mixtures: application to simulated datasets and real data from the Navajo Birth Cohort Study
Source: Environ Health. 2019 May 9;18:46. doi: 10.1186/s12940-019-0482-6 (PMC6507239; doi:10.1186/s12940-019-0482-6)
Supplement: Supplementary file 1 — Table S1. Summary statistics for oxidative stress prostaglandin ratio biomarker by demographic characteristics. Figure S1. Correlation matrix among metals in the NBCS dataset. The pairwise correlation between the metals as measured by the Pearson’s r between log transformed metal exposures are shown in the figure below, color coded by the magnitude of the correlation. Figure S2. Distribution of the metals in the NBCS dataset before and after standardization are described and compared using boxplots. Table S2. Simulated Data from the NIEHS Workshop (No Interaction). Table S3. Simulated Data from the NIEHS Workshop (Including Pairwise Interaction Terms). Table S4. Analysis of Local Simulated Dataset 2 (No Interaction). Table S5. Analysis of Local Simulated Dataset 2 (With Interaction). Figure S3. Analysis of Local Simulated Dataset (No Interaction, rho=0.3). Table S6. Performance Evaluation for Each Approach for Simulated Dataset (No Interaction, rho=0.3). Figure S4. Analysis of Local Simulated Dataset (With Interaction, rho=0.3). Table S7. Performance Evaluation of Each Approach for Analysis of Local Simulated Dataset (With Interaction, rho=0.3). Figure S5. Analysis of Local Simulated Dataset (No Interaction, rho=0.5). Table S8. Performance Evaluation for Each Approach for Simulated Dataset (No Interaction, rho=0.5). Figure S6. Analysis of Local Simulated Dataset (With Interaction, rho=0.5). Table S9. Performance Evaluation of Each Approach for Analysis of Local Simulated Dataset (With Interaction, rho=0.5). Figure S7. Analysis of Local Simulated Dataset (No Interaction, rho=0.7). Table S10. Performance Evaluation for Each Approach for Simulated Dataset (No Interaction, rho=0.7). Figure S8. Analysis of Local Simulated Dataset (With Interaction, rho=0.7). Table S11. Performance Evaluation of Each Approach for Analysis of Local Simulated Dataset (With Interaction, rho=0.7). Figure S9. Analysis of Local Simulated Dataset (No Interaction, rho=0.8). Table S12. Performance [file 12940_2019_482_MOESM1_ESM.docx]

Table S1. Summary statistics for oxidative stress prostaglandin ratio biomarker by demographic characteristics.

| **Characteristic** | **Category** | **n (%)** | **Median [IQR]** | ***p*** |
| --- | --- | --- | --- | --- |
| Zinc level | High | 66 (50.0) | 0.258 [0.184 - 0.310] | 0.32 ^a^ |
|  | Low | 66 (50.0) | 0.240 [0.183 - 0.301] |  |
| Education above high school | No | 65 (56.0) | 0.250 [0.184 - 0.313] | 0.14 ^a^ |
|  | Yes | 51 (44.0) | 0.240 [0.160 - 0.296] |  |
| Annual household income <$20,000 | No | 41 (41.8) | 0.256 [0.176 - 0.302] | 0.97 ^a^ |
|  | Yes | 57 (58.2) | 0.229 [0.183 - 0.311] |  |
| Currently unemployed | No | 42 (35.6) | 0.283 [0.226 - 0.320] | **0.04** ^a^ |
|  | Yes | 76 (64.4) | 0.210 [0.162 - 0.296] |  |
| Alcohol usage in the past year | No | 84 (71.2) | 0.240 [0.184 - 0.307] | 0.88 ^a^ |
|  | Yes | 34 (28.8) | 0.253 [0.169 - 0.301] |  |
| Ceremonial tobacco usage | No | 73 (61.9) | 0.230 [0.166 - 0.300] | **0.05** ^a^ |
|  | Yes | 45 (38.1) | 0.275 [0.188 - 0.307] |  |
| Vitamin usage | No | 39 (33.1) | 0.256 [0.185 - 0.304] | 0.83 ^a^ |
|  | Yes | 79 (66.9) | 0.240 [0.171 - 0.309] |  |
| Wood used for home heating | No | 48 (40.7) | 0.259 [0.180 - 0.303] | 0.85 ^a^ |
|  | Yes | 70 (59.3) | 0.238 [0.178 - 0.308] |  |
| Coal used for home heating | No | 90 (76.3) | 0.250 [0.183 - 0.306] | 0.94 ^a^ |
|  | Yes | 28 (23.7) | 0.226 [0.170 - 0.313] |  |
| BMI | Normal | 28 (28.9) | 0.256 [0.185 - 0.311] | 0.46 ^b^ |
|  | Overweight | 37 (38.1) | 0.229 [0.161 - 0.292] |  |
|  | Obese | 32 (33.0) | 0.264 [0.175 - 0.300] |  |
| Trimester stage | 1st | 25 (18.9) | 0.296 [0.273 - 0.324] | **0.0007** ^b^ |
|  | 2nd | 59 (44.7) | 0.252 [0.192 - 0.300] |  |
|  | 3rd | 48 (36.4) | 0.203 [0.160 - 0.287] |  |

Abbreviations: BMI, body mass index

a P-values were calcuclated using two - sample t tests of the oxidative stress prostaglandin ratio biomarker.

b P-values were calcuclated using the one-way analysis of variance F tests of the oxidative stress prostaglandin ratio biomarker.

Figure S1. Correlation matrix among metals in the NBCS dataset. The pairwise correlation between the metals as measured by the Pearson’s r between log transformed metal exposures are shown in the figure below, color coded by the magnitude of the correlation.


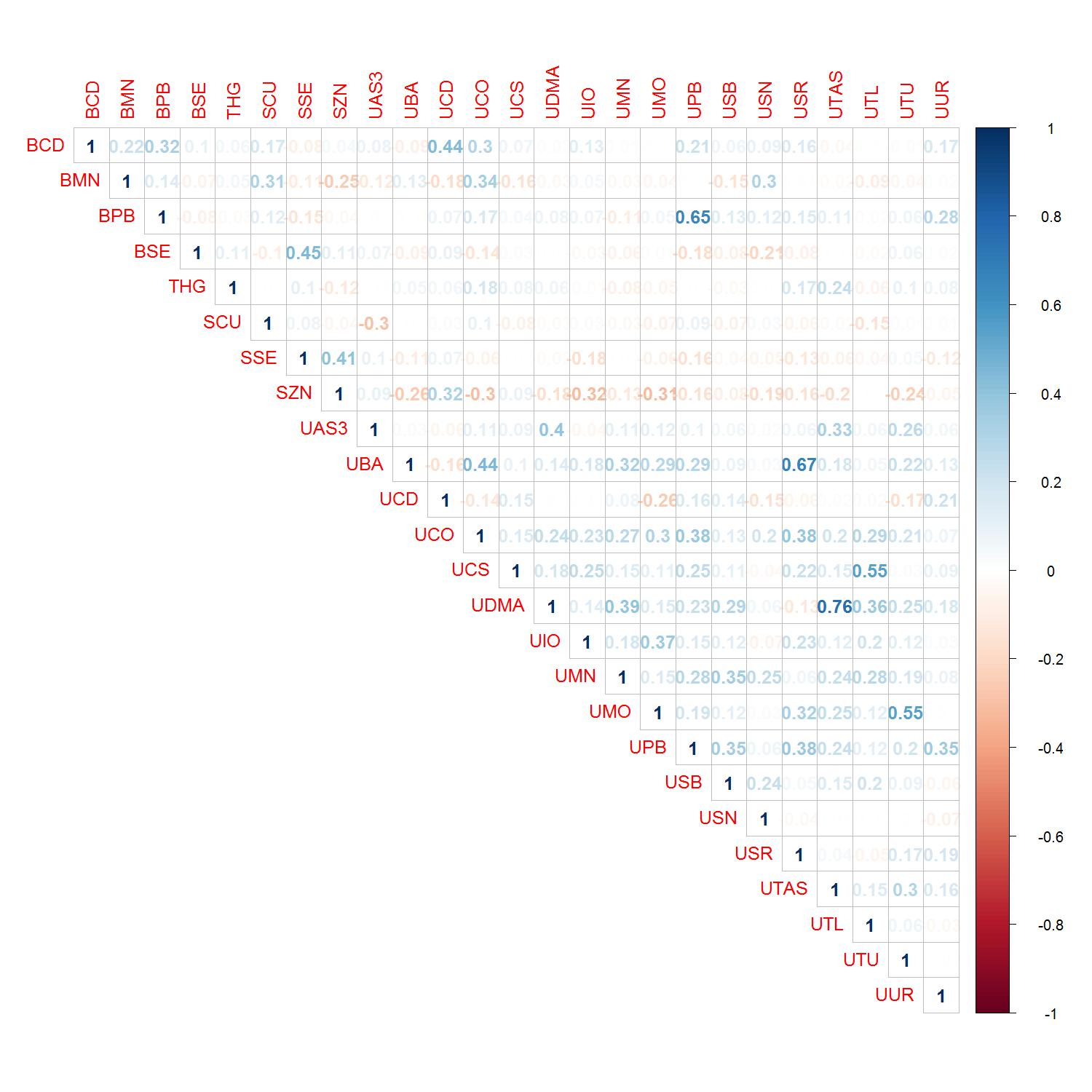


Figure S2. Distribution of the metals in the NBCS dataset before and after standardization are described and compared using boxplots.


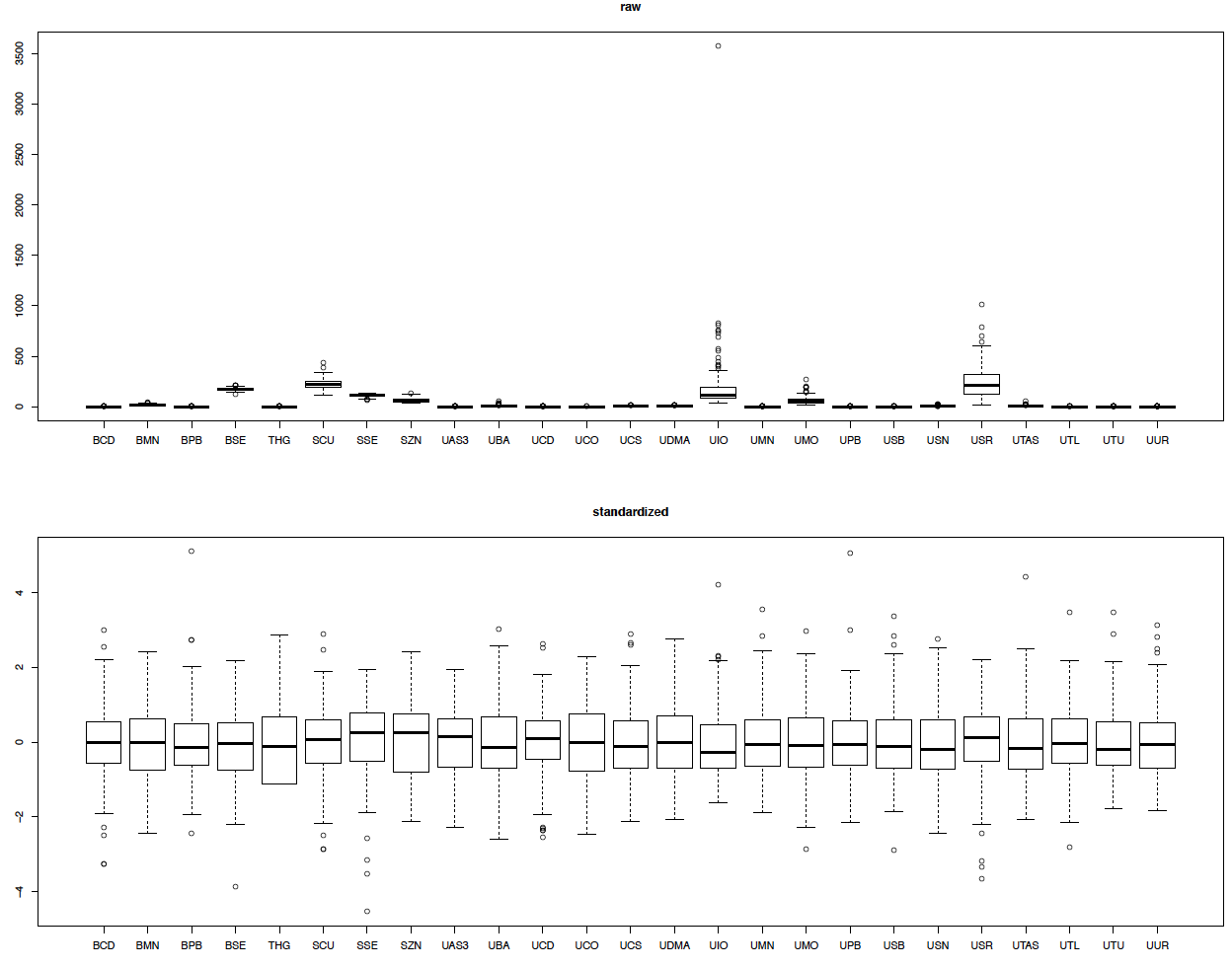


Table S2. Simulated Data from the NIEHS Workshop (No Interaction)

| True Model | Adaptive lasso | | CART+ Adaptive lasso | | RF+ Adaptive lasso | |
| --- | --- | --- | --- | --- | --- | --- |
|  | Selected Xs | $\hat{\beta}$ (SE) | Selected Xs | $\hat{\beta}$ (SE) | Selected Xs | $\hat{\beta}$ (SE) |
| X1 (+) | X1 | 3.3(0.41) * | X1 | 3.4 (0.39) * | X1 | 3.5(0.26) * |
| X2 (+) | X2 | 0.89(0.7) |  |  |  |  |
|  | X3 | 0.095(0.38) |  |  |  |  |
| X4 (-) | X4 | -1(0.22) * |  |  |  |  |
| X5 (-) | X5 | -4.1(0.16) * |  |  | X5 | -4(0.16) * |
| X7 (+) | X7 | 3.7(0.16) * | X7 | 3.4(0.24) * | X7 | 3.8(0.16) * |
|  | Z | 12(0.46) * | Z | 12(0.7) * | Z | 12(0.46) * |

Xs: Covariates that were selected to be associated with outcome Y.

$\hat{\beta}$ (SE) : Estimated regression coefficients (Standard errors for the regression coefficients).

*: The coefficients are significantly different from zero.

Dataset was simulated from a biologically-based dose response function: $Y=f\left[ X_{1},X_{2},X_{4},X_{5},X_{7} \right]+\gamma Z+\epsilon$ (https://www.niehs.nih.gov/news/events/pastmtg/2015/statistical/index.cfm).

Table S3. Simulated Data from the NIEHS Workshop (Including Pairwise Interaction Terms).

| Adaptive lasso | | CART+ Adaptive lasso | | RF+ Adaptive lasso | |
| --- | --- | --- | --- | --- | --- |
|  | $\hat{\beta}$ (SE) |  | $\hat{\beta}$ (SE) |  | $\hat{\beta}$ (SE) |
| X1 | 3.2(0.57) * | X1 | 3.2(0.92) * | X1 | 3.1(0.62) * |
| X2 | 2(0.62) * |  |  | X2 | 1.5(0.65) * |
| X3 | 0.39(0.34) | X3 | 0.64(0.5) | X3 | 0.2(0.37) |
| X4 | -0.77(0.28) * |  |  |  |  |
| X5 | -4.2(0.25) * |  |  | X5 | -3.7(0.31) * |
| X6 | -0.29(0.22) |  |  |  |  |
| X7 | 3.7(0.14) * | X7 | 2.8(0.29) * | X7 | 3.8(0.28) * |
| Z | 9.9(0.42) * | Z | 10(0.73) * | Z | 10(0.47) * |
| X1:X3 | 0.65(0.24) * | X1:X3 | 0.79(0.4) * | X1:X3 | 0.54(0.25) * |
|  |  |  |  | X1:X5 | 0.35(0.27) |
| X1:X7 | 0.37(0.29) |  |  | X1:X7 | 0.62(0.25) * |
| X1:Z | 2.3(0.88) * | X1:Z | 2.6(1.5) | X1:Z | 2.3(0.96) * |
| X2:X7 | 0.88(0.56) |  |  |  |  |
| X3:X6 | -0.21(0.19) |  |  |  |  |
| X4:X5 | 0.88(0.2) * |  |  |  |  |
| X4:X7 | -0.48(0.21) * |  |  |  |  |
| X4:Z | -0.56(0.42) |  |  |  |  |
| X5:X6 | -0.84(0.14) * |  |  |  |  |
| X5:X7 | -0.54(0.15) * |  |  | X5:X7 | -0.67(0.16) * |
| X5:Z | -0.52(0.31) |  |  | X5:Z | -0.81(0.49) |
|  |  | X7:Z | 1.3(0.46) * | X7:Z | -0.043(0.45) |

Xs: Covariates that were selected to be associated with outcome Y.

$\hat{\beta}$ (SE) : Estimated regression coefficients (Standard errors for the regression coefficients).

*: The coefficients are significantly different from zero.

Dataset was simulated from a biologically-based dose response function: $Y=f\left[ X_{1},X_{2},X_{4},X_{5},X_{7} \right]+\gamma Z+\epsilon$ (https://www.niehs.nih.gov/news/events/pastmtg/2015/statistical/index.cfm).

Table S4. Analysis of Local Simulated Dataset (No Interaction).

| True Model | | Adaptive lasso | | CART+ Adaptive lasso | | RF+ Adaptive lasso | |
| --- | --- | --- | --- | --- | --- | --- | --- |
| Variable | True Beta | Selected Xs | $\hat{\beta}$ (SE) | Selected Xs | $\hat{\beta}$ (SE) | Selected Xs | $\hat{\beta}$ (SE) |
| X1 | 0.2 | X1 | 0.28(0.047) | X1 | 0.3(0.047) | X1 | 0.28(0.047) |
| X2 | 0.3 | X2 | 0.31(0.045) | X2 | 0.3(0.045) | X2 | 0.31(0.045) |
| X3 | 0 | X3 | -0.1(0.048) | X3 | -0.1(0.047) |  |  |
| X7 | 0 | X7 | 0.065(0.047) | X7 | 0.064(0.047) |  |  |
| X9 | -0.15 | X9 | -0.11(0.046) | X9 |  | X9 | -0.12(0.045) |
| X12 | 0.2 | X12 | 0.31(0.046) | X12 | 0.31(0.046) | X12 | 0.3(0.046) |
| X15 | 0.10 | X15 | 0.13(0.047) | X15 |  | X15 | 0.13(0.047) |
| X16 | -0.25 | X16 | -0.26(0.047) | X16 | -0.27(0.047) | X16 | -0.26(0.047) |
| X1*X12 | 0.1 |  |  |  |  |  |  |
|  |  |  |  |  |  |  |  |

Xs: Covariates that were selected to be associated with outcome Y.

$\hat{\beta}$ (SE) : Estimated regression coefficients (Standard errors for the regression coefficients).

Dataset was simulated from a function:

$Y=f\left[ \mathbf{X} \right]+\epsilon={0.2X}_{1}+0.3X_{2}-0.15X_{9}+0.2X_{12}+0.1X_{15}-0.25X_{16}+0.1X_{1}\times X_{12}+\epsilon$

Table S5. Analysis of Local Simulated Dataset (With Interaction).

| Adaptive lasso | | CART+ Adaptive lasso | | RF+ Adaptive lasso | |
| --- | --- | --- | --- | --- | --- |
| Selected Xs | $\hat{\beta}$ (SE) | Selected Xs | $\hat{\beta}$ (SE) | Selected Xs | $\hat{\beta}$ (SE) |
|  |  | X1 | 0.2(0.078) | X1 | 0.17(0.084) |
|  |  | X2 | 0.21(0.074) | X2 | 0.27(0.058) |
|  |  |  |  | X12 | 0.24(0.087) |
|  |  | X16 | -0.062(0.08) | X16 | -0.13(0.066) |
| X1:X2 | 0.015(0.034) |  |  |  |  |
| X1:X11 | 0.041(0.029) |  |  |  |  |
|  |  | X1:X7 | 0.0088(0.041) | X1:X7 | 0.017(0.042) |
| X1:X12 | 0.1(0.033) | X1:X12 | 0.081(0.038) | X1:X12 | 0.052(0.045) |
| X1:X15 | 0.066(0.035) |  |  | X1:X15 | 0.032(0.037) |
| X2:X10 | 0.043(0.032) |  |  | X2:X10 | 0.041(0.033) |
| X2:X14 | 0.074(0.036) | X2:X14 | 0.044(0.039) |  |  |
| X2:X18 | 0.081(0.035) |  |  |  |  |
| X2:X20 | 0.066(0.035) | X2:X20 | 0.049(0.04) |  |  |
| X3:X16 | -0.052(0.032) | X3:X16 | -0.039(0.036) |  |  |
|  |  | X3:X17 | -0.065(0.036) |  |  |
| X6:X9 | -0.055(0.033) |  |  | X6:X9 | -0.062(0.034) |
| X15:X6 | 0.056(0.032) |  |  | X15:X6 | 0.084(0.033) |
| X12:X7 | 0.072(0.028) | X12:X7 | 0.058(0.035) | X12:X7 | 0.056(0.038) |
| X16:X8 | -0.033(0.029) | X16:X8 | -0.048(0.03) | X16:X8 | -0.063(0.03) |
| X14:X9 | -0.051(0.033) |  |  |  |  |
| X10:X16 | -0.042(0.035) |  |  | X10:X16 | -0.059(0.037) |
| X12:X17 | 0.065(0.029) | X12:X17 | 0.1(0.035) |  |  |
| X14:X16 | -0.05(0.037) | X14:X16 | -0.07(0.038) |  |  |
| X18:X16 | -0.05(0.032) |  |  |  |  |
| X20:X16 | -0.046(0.036) | X20:X16 | -0.057(0.04) |  |  |
|  |  | X20:X12 | 0.038(0.038) |  |  |
|  |  |  |  | X12:X9 | -0.06(0.036) |

Xs: Covariates that were selected to be associated with outcome Y.

$\hat{\beta}$ (SE) : Estimated regression coefficients (Standard errors for the regression coefficients).

Dataset was simulated from a function:

$Y=f\left[ \mathbf{X} \right]+\epsilon={0.2X}_{1}+0.3X_{2}-0.15X_{9}+0.2X_{12}+0.1X_{15}-0.25X_{16}+0.1X_{1}\times X_{12}+\epsilon$

Figure S3. Analysis of Local Simulated Dataset (No Interaction, rho=0.3).


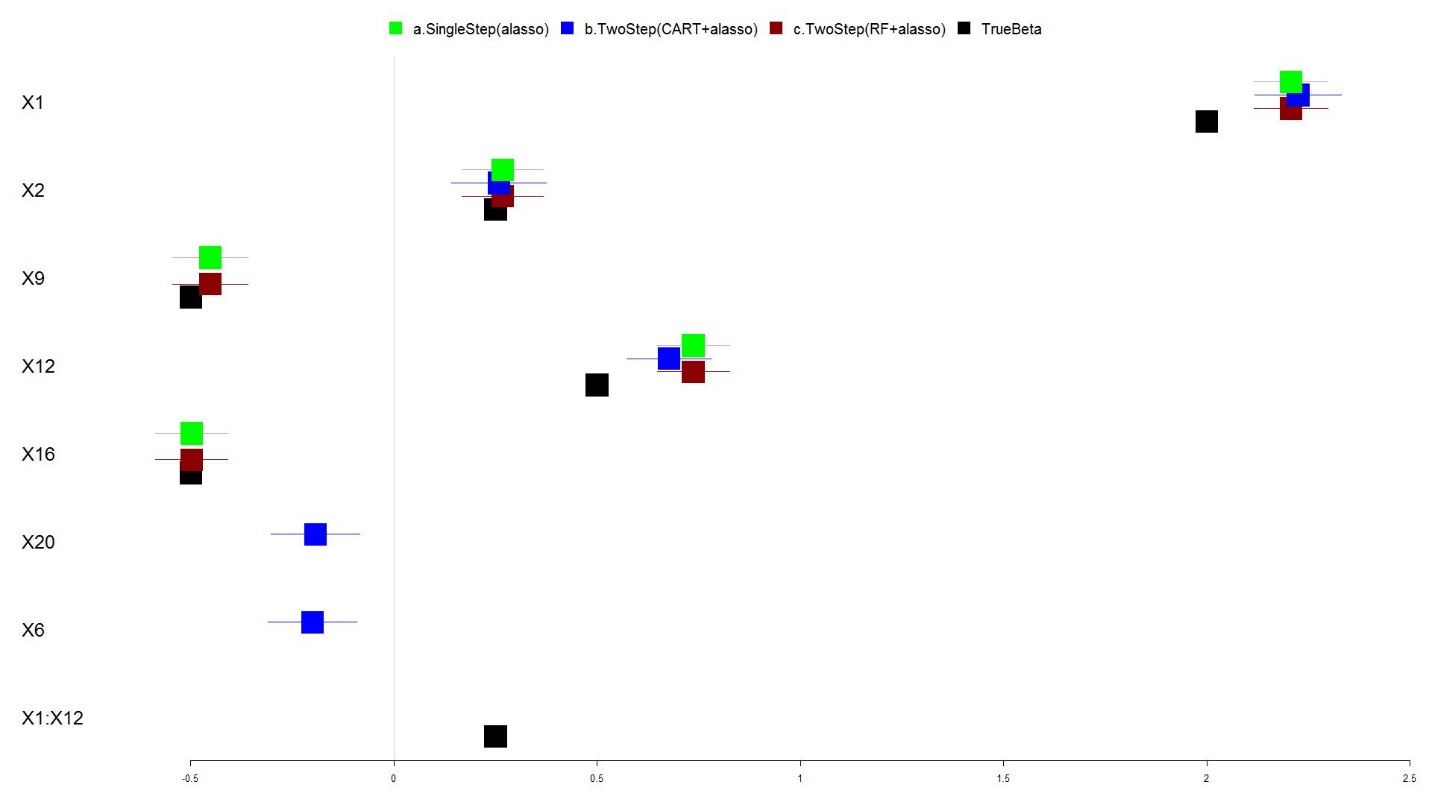

The forest plot describing the regression coefficients (boxes) and their 95% confidence intervals (lines) for modeling the relationship between exposure variables and outcome using three methods: (a) single step adaptive lasso (green); (b) two-step with CART followed by adaptive lasso approach (blue); and (c) two-step with RF followed by adaptive lasso approach (red). The black boxes indicate the true parameter coefficients used in the simulation. The numbers on X axis represent the magnitude of the regression coefficients.

Table S6. Performance Evaluation for Each Approach for Simulated Dataset (No Interaction, rho=0.3).

|  | Adaptive lasso | CART+ Adaptive lasso | RF+ Adaptive lasso |
| --- | --- | --- | --- |
| R^2^ | 0.861 | 0.811 | 0.861 |
| Adjusted R^2^ | 0.859 | 0.809 | 0.859 |
| MSE | 1.054 | 1.433 | 1.054 |
| MSE.CV | 1.050 | 1.429 | 1.052 |
| MSPE.CV | 1.092 | 1.484 | 1.082 |

Figure S4. Analysis of Local Simulated Dataset (With Interaction, rho=0.3).
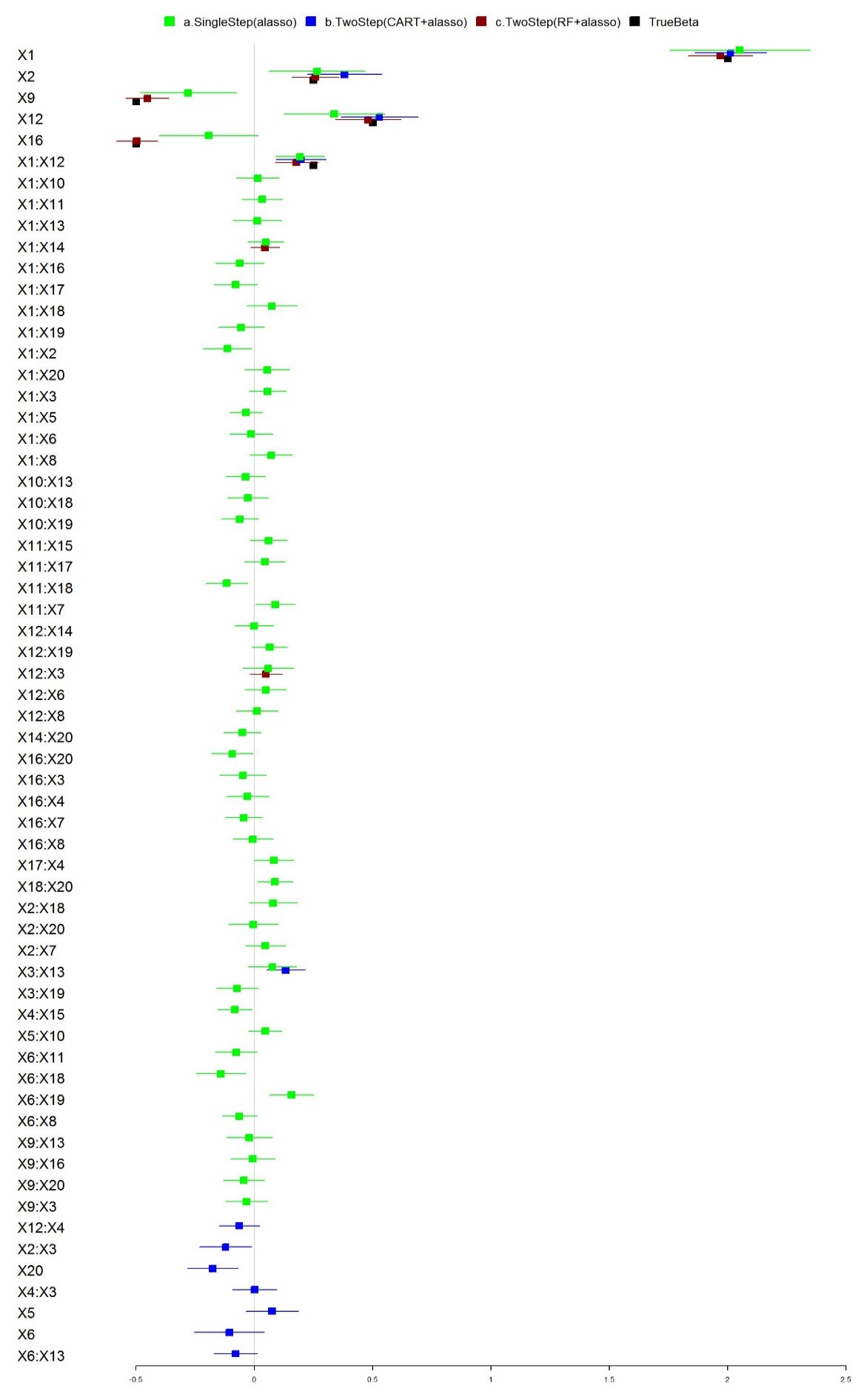


The forest plot describing the regression coefficients (boxes) and their 95% confidence intervals (lines) for modeling the relationship between exposure variables and outcome using three methods: (a) single step adaptive lasso (green); (b) two-step with CART followed by adaptive lasso approach (blue); and (c) two-step with RF followed by adaptive lasso approach (red). The black boxes indicate the true parameter coefficients ($\hat{\beta}$) used in the simulation. The numbers on X axis represent the magnitude of the regression coefficients.

Table S7. Performance Evaluation of Each Approach for Analysis of Local Simulated Dataset (With Interaction, rho=0.3).

|  | Adaptive lasso | CART+ Adaptive lasso | RF+ Adaptive lasso |
| --- | --- | --- | --- |
| R^2^ | 0.890 | 0.821 | 0.867 |
| Adjusted R^2^ | 0.876 | 0.817 | 0.865 |
| MSE | 0.832 | 1.353 | 1.004 |
| MSE.CV | 0.824 | 1.323 | 0.998 |
| MSPE.CV | 1.322 | 1.559 | 1.067 |

Figure S5. Analysis of Local Simulated Dataset (No Interaction, rho=0.5).


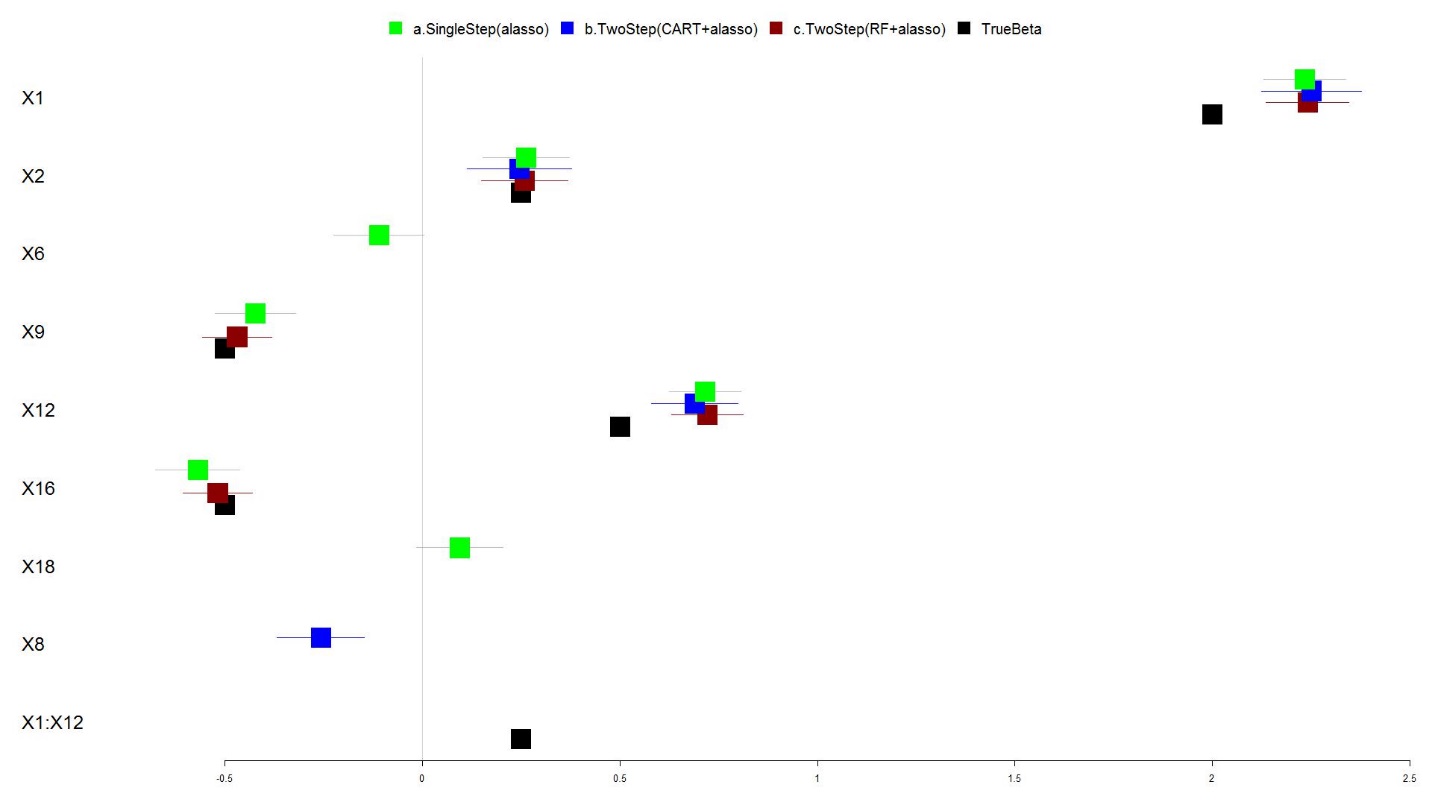

The forest plot describing the regression coefficients (boxes) and their 95% confidence intervals (lines) for modeling the relationship between exposure variables and outcome using three methods: (a) single step adaptive lasso (green); (b) two-step with CART followed by adaptive lasso approach (blue); and (c) two-step with RF followed by adaptive lasso approach (red). The black boxes indicate the true parameter coefficients used in the simulation. The numbers on X axis represent the magnitude of the regression coefficients.

Table S8. Performance Evaluation for Each Approach for Simulated Dataset (No Interaction, rho=0.5).

|  | Adaptive lasso | CART+ Adaptive lasso | RF+ Adaptive lasso |
| --- | --- | --- | --- |
| R^2^ | 0.862 | 0.798 | 0.861 |
| Adjusted R^2^ | 0.860 | 0.797 | 0.859 |
| MSE | 1.057 | 1.549 | 1.070 |
| MSE.CV | 1.058 | 1.545 | 1.068 |
| MSPE.CV | 1.118 | 1.596 | 1.103 |

Figure S6. Analysis of Local Simulated Dataset (With Interaction, rho=0.5).


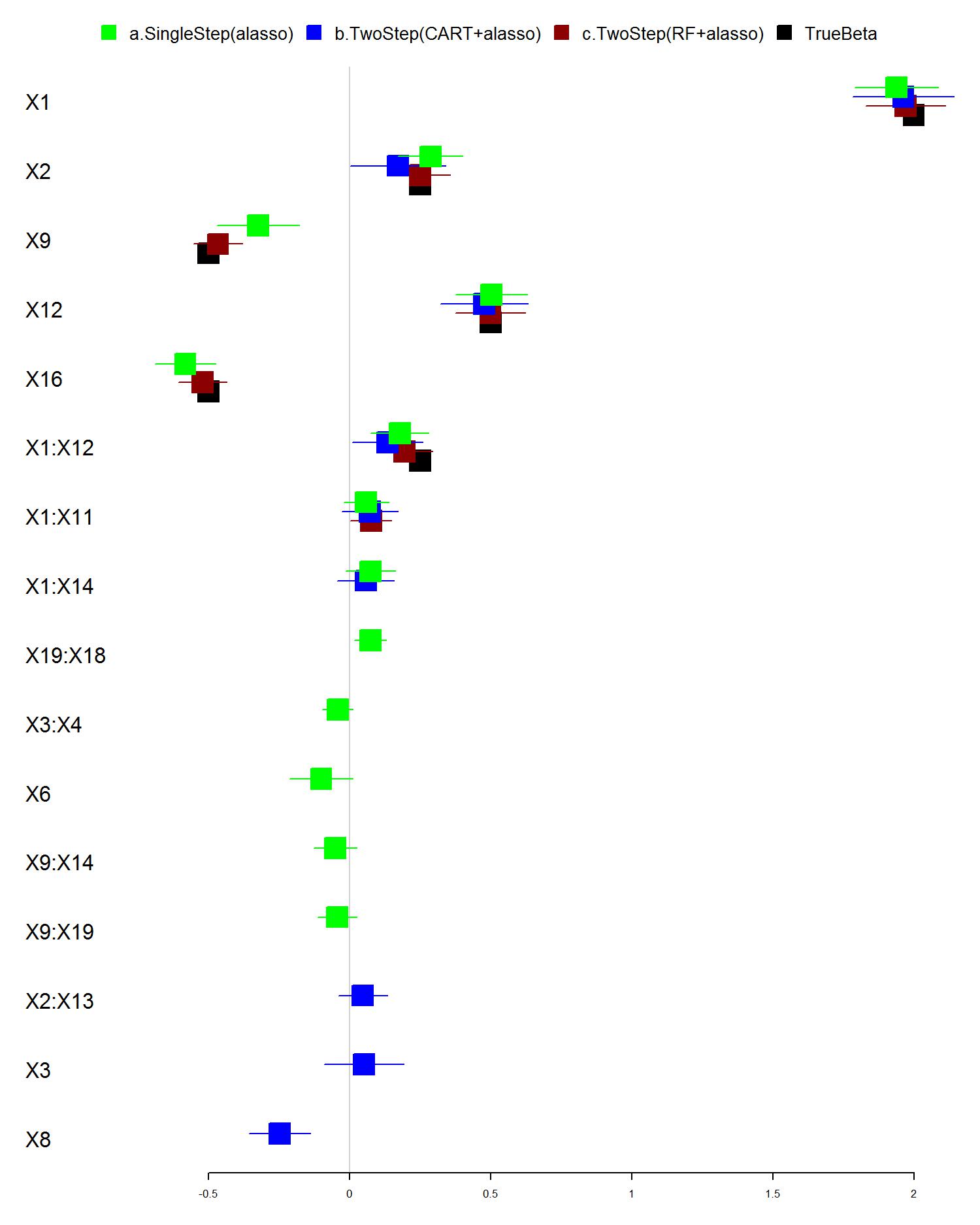


The forest plot describing the regression coefficients (boxes) and their 95% confidence intervals (lines) for modeling the relationship between exposure variables and outcome using three methods: (a) single step adaptive lasso (green); (b) two-step with CART followed by adaptive lasso approach (blue); and (c) two-step with RF followed by adaptive lasso approach (red). The black boxes indicate the true parameter coefficients ($\hat{\beta}$) used in the simulation. The numbers on X axis represent the magnitude of the regression coefficients.

Table S9. Performance Evaluation of Each Approach for Analysis of Local Simulated Dataset (With Interaction, rho=0.5).

|  | Adaptive lasso | CART+ Adaptive lasso | RF+ Adaptive lasso |
| --- | --- | --- | --- |
| R^2^ | 0.872 | 0.807 | 0.868 |
| Adjusted R^2^ | 0.869 | 0.803 | 0.866 |
| MSE | 0.980 | 1.482 | 1.010 |
| MSE.CV | 0.926 | 1.461 | 0.988 |
| MSPE.CV | 1.248 | 1.590 | 1.098 |

Figure S7. Analysis of Local Simulated Dataset (No Interaction, rho=0.7).


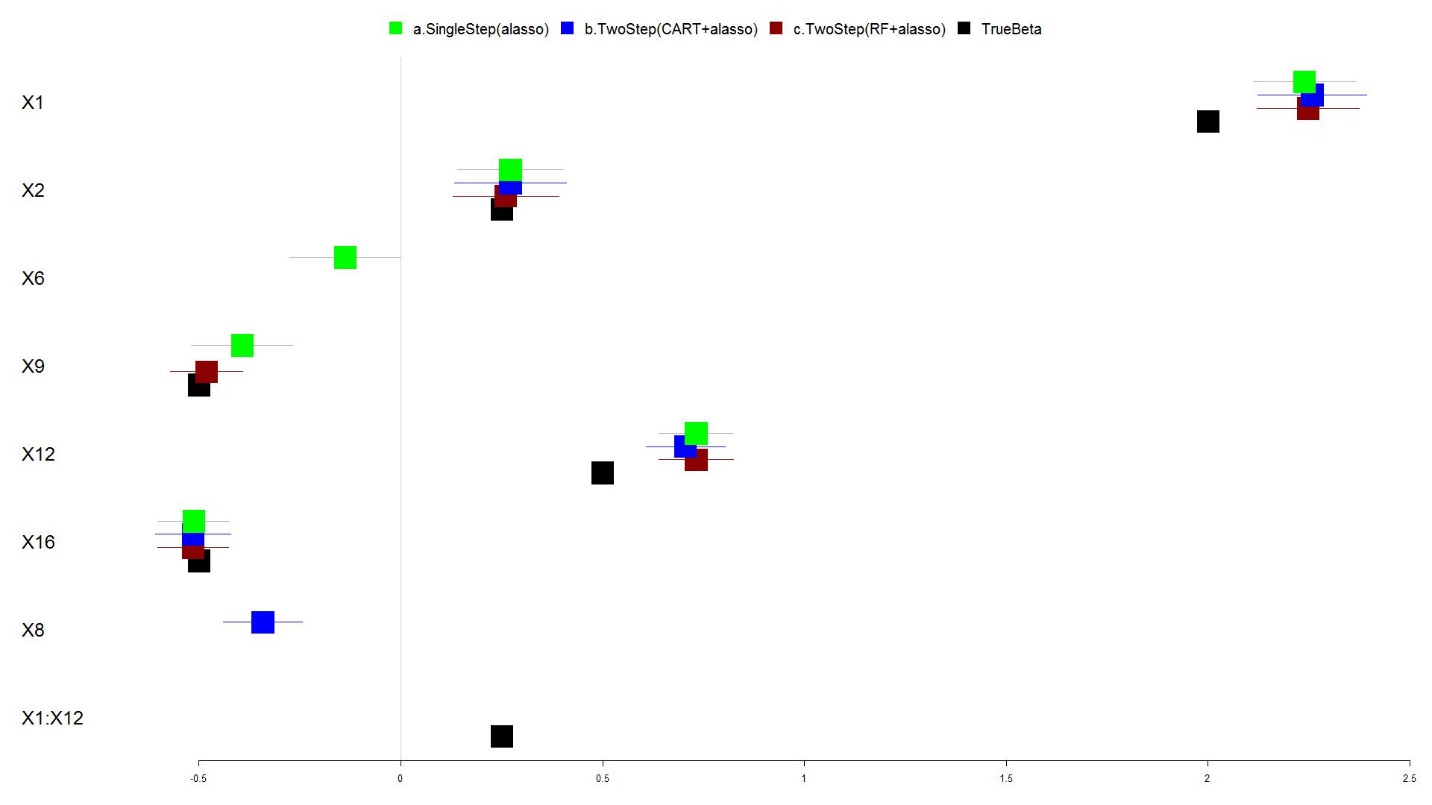


The forest plot describing the regression coefficients (boxes) and their 95% confidence intervals (lines) for modeling the relationship between exposure variables and outcome using three methods: (a) single step adaptive lasso (green); (b) two-step with CART followed by adaptive lasso approach (blue); and (c) two-step with RF followed by adaptive lasso approach (red). The black boxes indicate the true parameter coefficients used in the simulation. The numbers on X axis represent the magnitude of the regression coefficients.

Table S10. Performance Evaluation for Each Approach for Simulated Dataset (No Interaction, rho=0.7).

|  | Adaptive lasso | CART+ Adaptive lasso | RF+ Adaptive lasso |
| --- | --- | --- | --- |
| R^2^ | 0.865 | 0.848 | 0.864 |
| Adjusted R^2^ | 0.864 | 0.846 | 0.863 |
| MSE | 1.063 | 1.202 | 1.071 |
| MSE.CV | 1.059 | 1.197 | 1.069 |
| MSPE.CV | 1.116 | 1.242 | 1.103 |

Figure S8. Analysis of Local Simulated Dataset (With Interaction, rho=0.7).


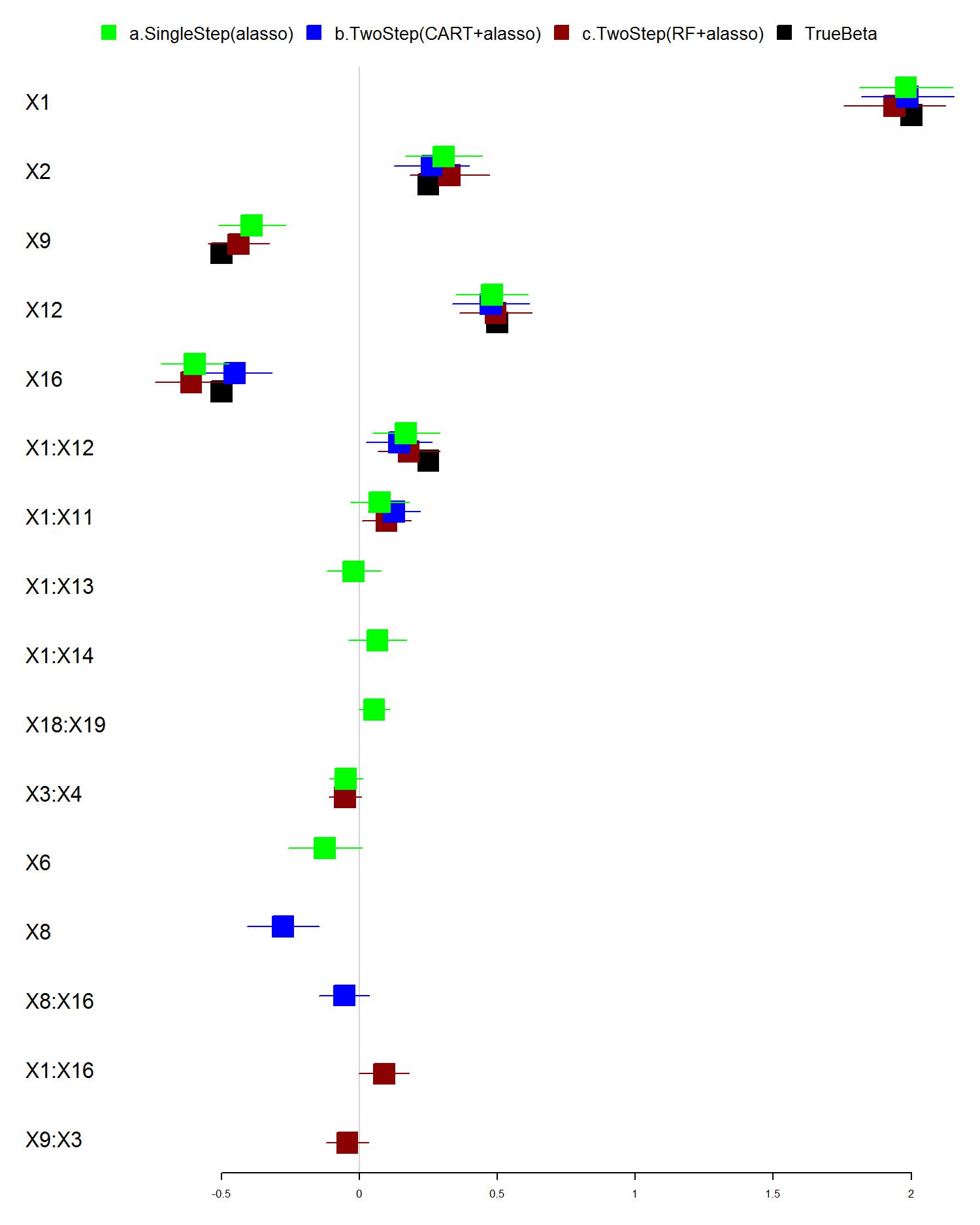


The forest plot describing the regression coefficients (boxes) and their 95% confidence intervals (lines) for modeling the relationship between exposure variables and outcome using three methods: (a) single step adaptive lasso (green); (b) two-step with CART followed by adaptive lasso approach (blue); and (c) two-step with RF followed by adaptive lasso approach (red). The black boxes indicate the true parameter coefficients ($\hat{\beta}$) used in the simulation. The numbers on X axis represent the magnitude of the regression coefficients.

Table S11. Performance Evaluation of Each Approach for Analysis of Local Simulated Dataset (With Interaction, rho=0.7).

|  | Adaptive lasso | CART+ Adaptive lasso | RF+ Adaptive lasso |
| --- | --- | --- | --- |
| R^2^ | 0.875 | 0.856 | 0.874 |
| Adjusted R^2^ | 0.872 | 0.854 | 0.871 |
| MSE | 0.989 | 1.136 | 0.996 |
| MSE.CV | 0.943 | 1.120 | 0.991 |
| MSPE.CV | 1.204 | 1.236 | 1.095 |

Figure S9. Analysis of Local Simulated Dataset (No Interaction, rho=0.8).


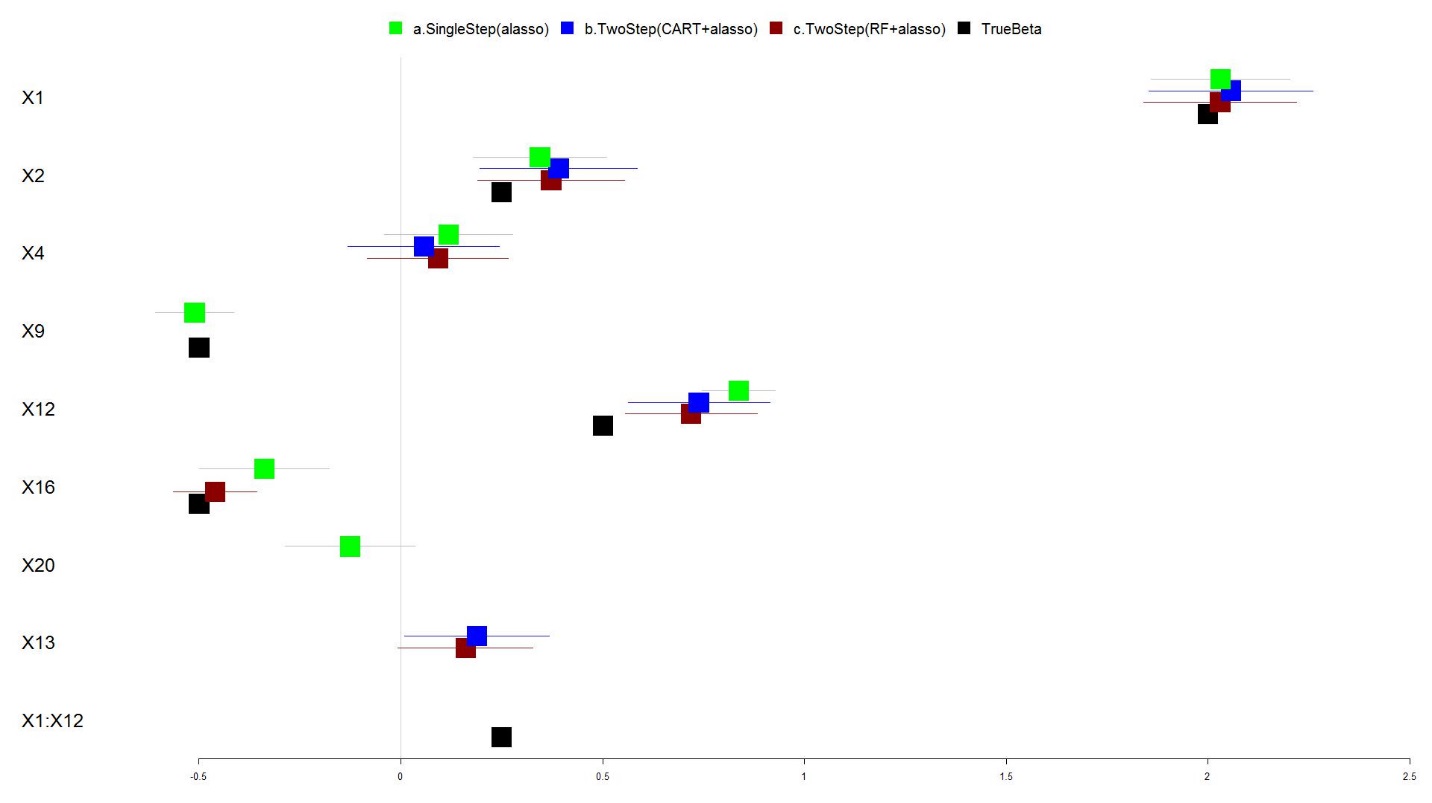


The forest plot describing the regression coefficients (boxes) and their 95% confidence intervals (lines) for modeling the relationship between exposure variables and outcome using three methods: (a) single step adaptive lasso (green); (b) two-step with CART followed by adaptive lasso approach (blue); and (c) two-step with RF followed by adaptive lasso approach (red). The black boxes indicate the true parameter coefficients used in the simulation. The numbers on X axis represent the magnitude of the regression coefficients.

Table S12. Performance Evaluation for Each Approach for Simulated Dataset (No Interaction, rho=0.8).

|  | Adaptive lasso | CART+ Adaptive lasso | RF+ Adaptive lasso |
| --- | --- | --- | --- |
| R^2^ | 0.870 | 0.816 | 0.841 |
| Adjusted R^2^ | 0.868 | 0.814 | 0.839 |
| MSE | 1.096 | 1.536 | 1.332 |
| MSE.CV | 1.096 | 1.537 | 1.332 |
| MSPE.CV | 1.141 | 1.552 | 1.370 |

Figure S10. Analysis of Local Simulated Dataset (With Interaction, rho=0.8).


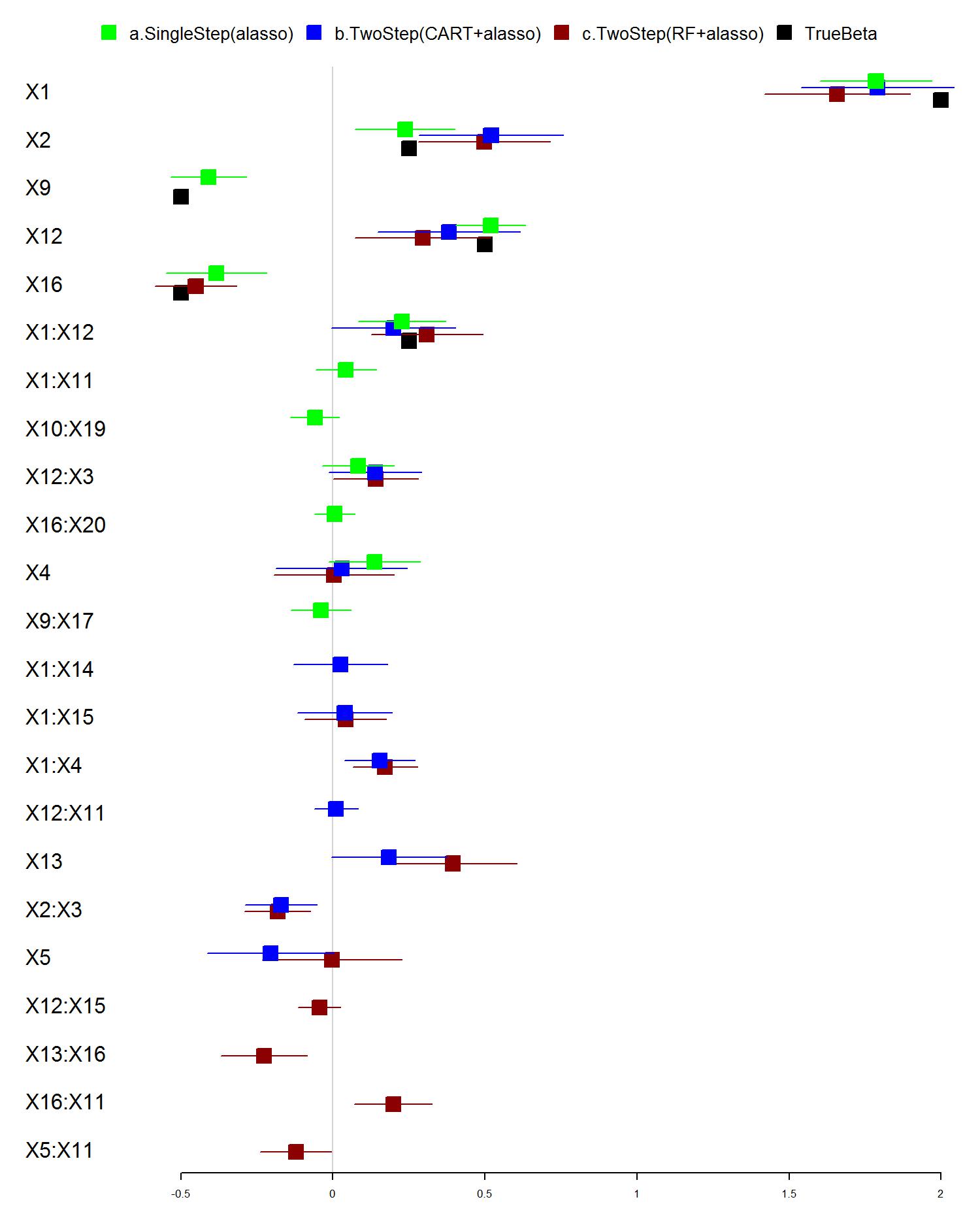


The forest plot describing the regression coefficients (boxes) and their 95% confidence intervals (lines) for modeling the relationship between exposure variables and outcome using three methods: (a) single step adaptive lasso (green); (b) two-step with CART followed by adaptive lasso approach (blue); and (c) two-step with RF followed by adaptive lasso approach (red). The black boxes indicate the true parameter coefficients ($\hat{\beta}$) used in the simulation. The numbers on X axis represent the magnitude of the regression coefficients.

Table S13. Performance Evaluation of Each Approach for Analysis of Local Simulated Dataset (With Interaction, rho=0.8).

|  | Adaptive lasso | CART+ Adaptive lasso | RF+ Adaptive lasso |
| --- | --- | --- | --- |
| R^2^ | 0.886 | 0.838 | 0.867 |
| Adjusted R^2^ | 0.883 | 0.834 | 0.862 |
| MSE | 0.967 | 1.374 | 1.141 |
| MSE.CV | 0.962 | 1.366 | 1.142 |
| MSPE.CV | 1.060 | 1.496 | 1.309 |

Table S14. Association analyses of the metal exposures and oxidative stress biomarkers collected in the NBCS study (No Interaction).

| Adaptive lasso | | CART+ Adaptive lasso | | RF+ Adaptive lasso | |
| --- | --- | --- | --- | --- | --- |
| Variable | $\hat{\beta}$ (SE) | Variable | $\hat{\beta}$ (SE) | Variable | $\hat{\beta}$ (SE) |
| Ceremonial  Tobacco | 0.046(0.015) * |  |  | CeremonialTobacco | 0.045(0.015) * |
| Trimester | -0.026(0.011) * |  |  | Trimester | -0.028(0.011) * |
| UAS3 | 0.0054(0.0081) | UAS3 | 0.0095(0.0086) |  |  |
| UBA | -0.022(0.0079) * |  |  | UBA | -0.021(0.0078) * |
| UCS | 0.019(0.009) * |  |  | UCS | 0.019(0.0089) * |
| UDMA | 0.023(0.012) | UDMA | 0.018(0.013) | UDMA | 0.026(0.011) * |
| UTAS | 0.0084(0.01) | UTAS | 0.0093(0.012) | UTAS | 0.0082(0.01) |
| UTL | -0.045(0.0096) * | UTL | -0.028(0.0089) * | UTL | -0.046(0.0095) * |
|  |  | BMN | -0.011(0.0082) |  |  |
|  |  | SZN | 0.011(0.0085) |  |  |
|  |  | UCO | -0.0061(0.0091) |  |  |
|  |  | BPB | 0.0094(0.0076) |  |  |

$\hat{\beta}$ (SE) : Estimated regression coefficients (Standard errors) for the selected covariates associated with oxidative stress biomarkers.

Table S15. Association analyses of the metal exposures and oxidative stress biomarkers collected in the NBCS study (With Interaction).

| Adaptive lasso | | CART+ Adaptive lasso | | RF+ Adaptive lasso | |
| --- | --- | --- | --- | --- | --- |
| Variable | $\hat{\beta}$ (SE) | Variable | $\hat{\beta}$ (SE) | Variable | $\hat{\beta}$ (SE) |
| Age_Intvw | 0.0017(0.001) | BMN | -0.012(0.0075) | Ceremonial  Tobacco | 0.046(0.015) * |
| Trimester | -0.024(0.011) * | SZN | 0.0096(0.008) | Trimester | -0.025(0.011) * |
| BMN | -0.0068(0.0066) | UAS3 | 0.0055(0.0079) | UAS3 | 0.0067(0.0083) |
| UAS3 | -0.0015(0.0075) | UTAS | 0.022(0.0082) * | UBA | -0.027(0.0078) * |
| UBA | -0.0061(0.0075) | UTL | -0.015(0.0082) | UCS | 0.02(0.0091) * |
| UDMA | 0.027(0.013) * | BMN:SCU | 0.012(0.0082) | UDMA | 0.018(0.013) |
| UTAS | -0.0049(0.011) | BPB:UDMA | 0.0074(0.009) | UIO | 0.013(0.0073) |
| UTL | -0.024(0.0074) * | BPB:UPB | 0.0034(0.0031) | UTAS | 0.0085(0.011) |
| BMN:USB | 0.012(0.0063) | UTL:BPB | -0.026(0.008) * | UTL | -0.044(0.0097) * |
| UTL:BPB | -0.028(0.0072) * | BSE:USB | 0.0067(0.0069) | SZN:UCO | 0.021(0.0085) * |
| BPB:UUR | 0.0052(0.0043) | SZN:SCU | -0.0041(0.0084) | UCS:SZN | -0.02(0.0077) * |
| USB:BSE | 0.016(0.0067) * | UTL:SCU | 0.0065(0.0079) | UDMA:SZN | -0.012(0.0091) |
| THG:UCO | -0.0049(0.0071) | SSE:UCO | 0.012(0.0083) | UTL:SZN | 0.0077(0.0093) |
| SCU:SZN | -0.000071 (0.0074) | UTL:SSE | 0.0022(0.0074) | UAS3:UCO | 0.0043(0.0097) |
| UCO:SCU | 0.016(0.008) | UAS3:UCO | 0.018(0.0094) | UAS3:UTAS | 0.015(0.0083) |
| UCO:SSE | 0.0048(0.0083) | UAS3:UTAS | -0.0018(0.0092) | UAS3:UTL | -0.017(0.008) * |
| SSE:UIO | 0.0061(0.0063) | UDMA:UCO | 0.012(0.0089) | UBA:UIO | 0.0096(0.0078) |
| UTL:SSE | -0.0006 (0.0067) | UTAS:USB | -0.0083(0.0077) | UBA:UTAS | -0.0096(0.0095) |
| UAS3:UCO | 0.014(0.0078) | UTAS:UTL | -0.009(0.0088) | UBA:UTL | -0.016(0.0095) |
| UCO:UCD | 0.0017(0.0081) |  |  | UDMA:UCO | 0.0077(0.0086) |
| UIO:UCD | 0.0092(0.007) |  |  | UCS:UDMA | -0.0096(0.0088) |
| UCD:USN | 0.0035(0.0058) |  |  | UCS:UTL | 0.011(0.0089) |
| UTAS:UCD | 0.015(0.0073) * |  |  | UIO:UTAS | 0.011(0.011) |
| UTL:UCD | 0.013(0.0088) |  |  | UTL:UMO | -0.0039(0.008) |
| UDMA:UCO | 0.0062(0.0076) |  |  | UTAS:UTL | -0.011(0.01) |
| UUR:UCO | 0.014(0.0065) * |  |  |  |  |
| UCS:USR | 0.02(0.0067) * |  |  |  |  |
| UDMA:USB | -0.011(0.01) |  |  |  |  |
| USN:UMN | -0.013(0.0056) * |  |  |  |  |
| UTAS:USB | 0.0066(0.01) |  |  |  |  |
| UTAS:UTL | -0.013(0.008) |  |  |  |  |

$\hat{\beta}$ (SE) : Estimated regression coefficients (Standard errors) for the selected covariates associated with oxidative stress biomarkers.
